# Co‐Targeting Plk1 and DNMT3a in Advanced Prostate Cancer

**DOI:** 10.1002/advs.202101458

**Published:** 2021-05-29

**Authors:** Zhuangzhuang Zhang, Lijun Cheng, Qiongsi Zhang, Yifan Kong, Daheng He, Kunyu Li, Matthew Rea, Jianling Wang, Ruixin Wang, Jinghui Liu, Zhiguo Li, Chongli Yuan, Enze Liu, Yvonne N. Fondufe‐Mittendorf, Lang Li, Tao Han, Chi Wang, Xiaoqi Liu

**Affiliations:** ^1^ Department of Toxicology and Cancer Biology University of Kentucky Lexington KY 40536 USA; ^2^ Department of Biomedical Informatics The Ohio State University Columbus OH 43210 USA; ^3^ Markey Cancer Center University of Kentucky Lexington KY 40536 USA; ^4^ Department of Molecular and Cellular Biochemistry University of Kentucky Lexington KY 40536 USA; ^5^ School of Chemical Engineering Purdue University West Lafayette IN 47907 USA

**Keywords:** autophagy, cell death, crosstalk, DNMT3a, PCa, phosphorylation, Plk1, prostate cancer

## Abstract

Because there is no effective treatment for late‐stage prostate cancer (PCa) at this moment, identifying novel targets for therapy of advanced PCa is urgently needed. A new network‐based systems biology approach, XDeath, is developed to detect crosstalk of signaling pathways associated with PCa progression. This unique integrated network merges gene causal regulation networks and protein‐protein interactions to identify novel co‐targets for PCa treatment. The results show that polo‐like kinase 1 (Plk1) and DNA methyltransferase 3A (DNMT3a)‐related signaling pathways are robustly enhanced during PCa progression and together they regulate autophagy as a common death mode. Mechanistically, it is shown that Plk1 phosphorylation of DNMT3a leads to its degradation in mitosis and that DNMT3a represses Plk1 transcription to inhibit autophagy in interphase, suggesting a negative feedback loop between these two proteins. Finally, a combination of the DNMT inhibitor 5‐Aza‐2’‐deoxycytidine (5‐Aza) with inhibition of Plk1 suppresses PCa synergistically.

## Graphical abstract

A unique approach, XDeath is developed to identify novel targets for advanced prostate cancer (PCa). While Polo‐like kinase 1 (Plk1) phosphorylation of DNA methyltransferase 3A (DNMT3a) leads to its degradation in mitosis, DNMT3a represses Plk1 transcription to inhibit autophagy in interphase. This negative feedback loop provides the rationale for a combination therapy to treat advanced PCa.

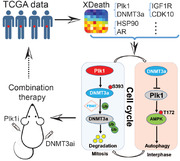

## 1 Introduction

Prostate cancer (PCa) is the most commonly diagnosed solid organ malignancy in men in the United States and remains the second leading cause of cancer death in this population.^[^ ^1^ ^]^ PCa progresses from intraepithelial neoplasia (benign), androgen‐dependent adenocarcinoma, to castration‐resistant prostate cancer (CRPC), a disease that is currently incurable.^[^ ^2^ ^]^ There is an urgent need to identify new targets whose inhibition can treat CRPC patients. It has been documented that cancer progression involves both activation of cell survival pathways and repression of cell‐death pathways. To date, the Nomenclature Committee on Cell Death (NCCD) has recognized an overall twelve classified cell‐death modes based on biochemical and cellular characteristics.^[^ ^3^ ^]^ It was shown that cell‐death proteins such as poly [ADP‐ribose] polymerase 1 (PARP)^[^ ^4^ ^]^ and bromodomain and extraterminal domain (BET)^[^ ^5^ ^]^ crosstalk with androgen receptor (AR) signaling, eventually contributing to PCa progression. However, the crosstalk mechanisms involved in cell growth and death that control prostate tumor cell aggressiveness remain largely elusive.

The histological evaluation of PCa is expressed in terms of Gleason score (GS), the grade of a tumor, and the most important prognostic factor in PCa.^[^ ^6^ ^]^ Cellular fate depends on the spatio‐temporal GS separation and integration of signaling processes with the variation of gene expression. Although gene signatures associated with GS were identified, switches of protein‐protein interaction driving the crosstalk architectural patterns in cell‐death signaling are still unknown.

Analysis of PCa‐related dynamic bio‐molecular interaction networks will improve our understanding of the complexity of the underlying molecular mechanisms during disease progression.^[^ ^7^ ^]^ Accordingly, systems biology approaches such as network‐based methods have been utilized to dissect the crosstalk mechanisms of signaling pathways in PCa.^[^ ^8^ ^]^ By integrating multiple expression datasets and protein interaction networks, one can identify functionally related gene modules within a gene community. Dissecting of such crosstalk modules associated with GS status are often used to identify potential signaling pathways and key proteins that could be targeted.^[^ ^9^ ^]^ Despite the advances in molecular technologies such as profiled PCa using RNA sequencing, which deepened our understanding of the genomic events responsible for the initial development and progression of PCa,^[^ ^9^ ^]^ a comprehensive molecular mechanism model(s) for studying PCa development is still lacking. Specifically, modulations of gene expression and biological processes that are related to cell death and that mediate important transitions during PCa progression remain to be defined in a systematic manner.

Polo‐like kinase 1 (Plk1) is involved in many aspects of the cell cycle, dysregulation of which is one common feature of cancer. Plk1 is overexpressed in PCa and linked to higher tumor grades, suggesting that Plk1 is involved in tumorigenesis and progression of PCa.^[^ ^10^ ^]^ DNA methyltransferase 3A (DNMT3a), which catalyzes DNA methylation of promoters of genes, is also elevated in PCa.^[^ ^11^ ^]^ Increased promoter methylation results in gene silencing of tumor suppressors. However, whether Plk1 and DNMT3a are coordinated to contribute to PCa progression is unknown.

Herein, we described the development of an interpretational computational network‐based approach, XDeath, to follow crosstalk of signaling pathways during PCa progression by taking advantage of the availability and integration of high‐throughput gene expression data and the genome‐wide protein‐protein interaction networks from STRING database (https://string‐db.org/). We have identified the crucial points in signaling crosstalk that are triggered by gene expression variations constant with an increase in GS. Analysis of twelve cell‐death modes has revealed novel synergistic co‐targets of PCa, especially in late‐stage PCa patients. Upon systematic analysis of the crosstalk mechanisms of cell‐death signaling pathways, we found that AR signaling was diminished, whereas Plk1 and DNMT3a signaling were activated during PCa progression. Quantification of crosstalk revealed a synergistic relationship of these two proteins, suggesting that they could be potential co‐targets for the treatment of advanced PCa. Mechanistically, we demonstrated that Plk1 phosphorylation of DNMT3a resulted in its degradation during mitosis and that DNMT3a‐associated inhibition of Plk1 transcription caused repression of autophagy in interphase. This supports the idea that a combination of Plk1 inhibition and the DNMT3a inhibitor 5‐Aza‐2’‐deoxycytidine (5‐Aza) will offer a new effective approach for PCa treatment.

## 2 Results

### 2.1 Identification of Hallmark Genes/Proteins Involved in PCa Progression

**Figure 1:**
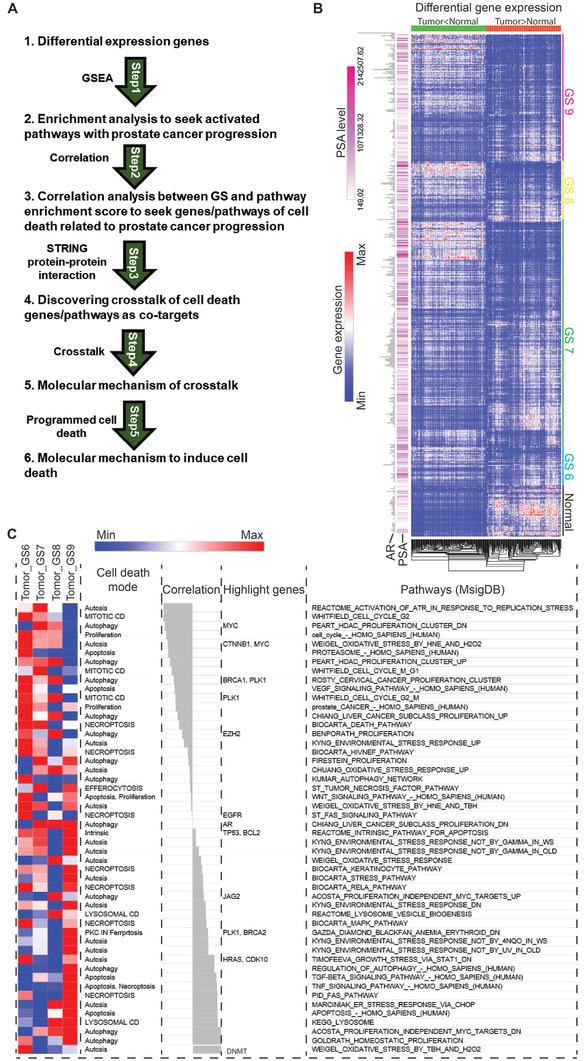
Establishment of a systems‐biology model to identify novel targets/pathways for PCa treatment. A) Framework of systematical detection of PCa progression with GS. B) Heat‐map of gene expression profile in 497 patients with different GSs. Top 1000 genes with significant expression differences were classified into two groups based on the levels of differentially expressed genes by comparing between normal with tumor tissues. C) Cell‐death modes involved in disease progression. NES is the gene enrichment score by GSEA analysis. All pathways are ranked by NES, then have a Pearson correlation analysis with Gleason Score.

**Figure 2:**
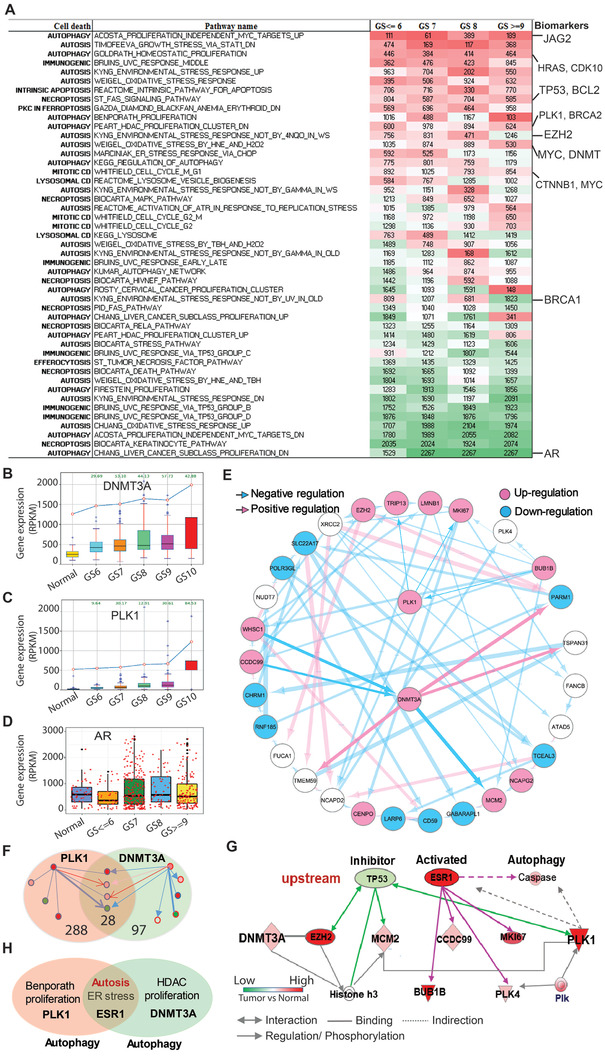
Enrichment analysis of cell death‐related pathways during PCa progression. A) Dynamic variation of pathways in 497 patients with various GSs. The numbers indicate the rank of indicated pathways changed during PCa progression. Green and red color indicates decreased and increased rank of corresponding pathway respectively. B) Increase in the level of DNMT3a during PCa progression. C) Increase in the level of Plk1 during PCa progression. D) AR expression variation during PCa progression. E) Bayesian network inference analysis to identify gene expression profiles in 497 PCa patients. Directed arcs represent causal dependencies and derive belief values by multiplying conditional probabilities. The thin line indicates low‐probability and thick line indicates high‐probability. The red color means the positive regulation and blue color is the negative regulation (inhibition). F) Crosstalk mechanism through the optimal path between Plk1 and DNMT3a identified by STRING PPI network analysis. The encoded nodal dependencies in BN enable it to predict accurately even when important values are unavailable in advanced patients (GS 8–10). The number of genes regulated by Plk1 and DNMT3a or regulating Plk1 and DNMT3a is 385, among which 288 and 97 genes are associated with Plk1 and DNMT3a respectively. There are 28 genes overlapping the regulation of PLK1 and DNMT3a. G) Plk1 and DNMT3a co‐expressing genes in 497 PCa patients were integrated with a causal gene regulation biology network. Causal analysis with Ingenuity Pathway Analysis (IPA, http://www.ingenuity.com) is used to infer and score regulator networks based on a large‐scale causal network. This casual network derives from the Ingenuity Integration Prior Knowledge Base, including 12 heterology networks. The analysis is to search for key genes or molecules contributing to the variation of Plk1 and DNMT3a in the gene regulation network in the advanced PCa patients from TCGA. Pearson correlation analysis is used to identify the co‐expression genes associated with Plk1 and DNMT3a with gene expression profiles of patients from TCGA. These co‐expression genesets are input into IPA to identify upstream regulators. In advantage of prior knowledge, the causal analysis approach can illuminate the biological activities that occurred in the crosstalk associated with the progression of PCa. H) Identification of estrogen receptor *α* and autophagy as a common signaling node shared by elevation of Plk1 and DNMT3a.

Using XDeath (**Figure** **1**A), we identified the top 400 high‐confidence genes out of 15 578 genes for PCa progression, which showed distinct spatio‐temporal expression patterns with different GSs. Here we named these genes as PCa progression hallmark genes (Figure 1B, Table S5, Supporting Information). These genes showed significantly differential expression between normal tissues and tumors (*p* < 0.05), and a strong correlation to GS value (correlation coefficient|*r*| > 0.5). Both positive correlation genes such as MYC, Plk1, and DNMT family genes and negative correlation genes including AR, EGFR, BRCA2, HRAS, CDK10, and JAG2 were identified (Figure 1C). Of note, AR activity gradually decreased with increasing GS until complete abolishment in the late stage of PCa (**Figure** **2**A, Table S6, Supporting Information).

Considering that prostate‐specific antigen (PSA) is a clinical biomarker for PCa screening, we also examined pattern space exhibition of the variations of gene expression accompanied with AR or PSA. The top 1000 genes with the largest standard deviation were selected from TCGA. Spearman correlation was used to calculate the expression correlation coefficient *r* of these genes with to be 0.35, 0.27, and 0.07 for PSA, AR, and GS, respectively, suggesting that GS did not have a significant contribution to the variations in the expression of these top 1000 genes, but PSA and AR did have had a strong correlation (Table S7, Supporting Information). AR (Figure S1A, Supporting Information) and PSA (Figure S1B, Supporting Information) exhibited variations for response to androgen deprivation therapy only temporarily, but not the disease progression represented by GS in pathology.

### 2.2 Enrichment Crosstalk of Cell‐Death Pathways During PCa Progression

We then identified the crosstalk of cell‐death pathways in 497 patients from TCGA by pathway enrichment analysis of the transcriptome of 550 gene expression profiles. 2267 out of 4367 matched pathways across 12 cell‐death modes were observed. Upon reconstruction of the dynamic cell‐death modes with increasing GS by XDeath, autophagy, apoptosis, proliferation, necroptosis, and ferroptosis were identified to be closely correlated with PCa progression (Figure 1C). The PCa progression hallmark pathways associated with cell death were identified by XDeath. Four of the top 10 pathways of cell death were related to the contribution of the cell cycle to PCa progression, among which Plk1 showed a strong correlation (Figure 1C). The detailed enrichment scores associated with GS in GSE21034 and TCGA are shown in Table S8, Supporting Information.

### 2.3 Potential Co‐Targets Identified for Late Stage PCa

By XDeath Bayesian gene inference analysis, we quantified the crosstalk to reveal the synergistic relationship of genes and pathways, and discovered potential co‐targets to treat advanced PCa by gene causal regulation network analysis. We identified a total of 126 hallmark genes/proteins and 35 pairs of genes/proteins involved in PCa progression, in which 7 pairs of genes and 15 single genes are recommended as druggable targets to treat PCa, such as target pairs of Plk1 and DNMT3a (Tables S9 and S10, Supporting Information). All target genes are significantly upregulated in tumors in comparison with the normal group. Then, we identified the top 3 pairs of genes, which are Plk1 and DNMT3a, heat‐shock protein 90 (HSP90) and AR, and insulin‐like growth factor‐1 receptor (IGF1R) and cyclin‐dependent kinase 10 (CDK10). In agreement, upregulation of both DNMT3a and Plk1 were observed in tumors with high GSs (Figure 2B,C, Figure S1C, Supporting Information). In addition, we also observed that AR aberrations in localized PCa did not typically involve AR itself (Figure 2D). These results suggest that AR might not be an ideal therapeutic target, especially in aggressive PCa. However, Plk1 and DNMT3a might be promising targets in late‐stage PCa when accumulating perturbations in active signaling pathways and crosstalk with each other alter gene regulation and pathways by causal relationship.

### 2.4 Crosstalk Between Plk1 and DNMT3a in the Gene Regulation Network

Multiple steps were performed to study the crosstalk between Plk1 and DNMT3a: 1) After Spearman correlation was used to construct the co‐expression network of Plk1 and DNMT3a, significant genes co‐expressed with Plk1 and DNMT3a were obtained by the threshold to correlation coefficient |*r*| > 0.5 and *p* value > 0.05 (Table S11, Supporting Information). 2) Bayesian network (BN) inference was used to reconstruct the gene regulation network between Plk1 and DNMT3a (Figure S1D, Supporting Information) based on the gene sets shown above, followed by identification of the causal direction of edges in the Plk1 and DNMT3a network (Figure 2E). To investigate the developmental programmed and response to Plk1 and DNMT3a targets on the gene regulation level, a GRN inference method, Fast and Furious Bayesian Network (FFBN), is used to characterize the transcriptional causal regulation of Plk1 and DNMT3a in advanced PCa (Figure 2E). BN represents causal dependencies (directed arcs) and derives belief values by multiplying conditional probabilities. Figure 2E describes the complex regulatory function network associated with Plk1 and DNMT3a. The encoded nodal dependencies in BN enable it to predict accurately even when important values are unavailable in advanced patients. 3) We then mapped edge weights (correlation coefficient *r*) and node weights (fold change of tumor vs normal tissues) of Plk1 and DNMT3a to the protein‐protein interaction (PPI) network (Figure 2F). Upon mapping the direction from causal gene regulation network to the PPI network to merge these two networks (Figure S1D, Supporting Information), Dijkstra's algorithm^[^ ^12^ ^]^ was used to seek the shortest path between Plk1 and DNMT3a (Figure S1D, Supporting Information) in the graph that the total sum of the vertice weights to be minimum as literature XTALK.^[^ ^13^ ^]^ 4) The result showed that DNMT3a regulated Plk1 by mediating EZH2, MCM2, CCDC99, MKI67, BUB1B, etc. (Figure 2G).

### 2.5 Upstream Analysis of Plk1/DNMT3a Crosstalk via IPA

The crosstalk between Plk1 and DNMT3a is the overlapping gene sets and the connecting edges between Plk1 and DNMT3a. These gene sets and their DEGs (55 normal tissues vs 497 tumors) *p*‐values were subjected to Ingenuity Pathway Analysis (IPA). The bioinformatics analysis^[^ ^14^ ^]^ revealed that both Plk1 and DNMT3a were involved in ESR1‐mediated autophagy and autosis, in which ESR1 was activated and TP53 was inactivated (Figure 2H). Gene sets crosstalk with DNMT3a and Plk1 identified by IPA upstream causal analysis are listed in Table S12, Supporting Information. Of note, that both Plk1‐ and DNMT3a‐regulated pathways are involved in autophagy suggests that autophagy likely contributes to PCa progression (Figure 2H). In agreement, both DNMT3a and Plk1 were upregulated in patients with metastatic CRPC by comparing profiles between 22 PCa benign tissues and 32 metastatic CRPC tissues to PDX002286 data analysis.

### 2.6 Identifying Methylation Aberrations of Genes Related to PCa Progression

**Figure 3:**
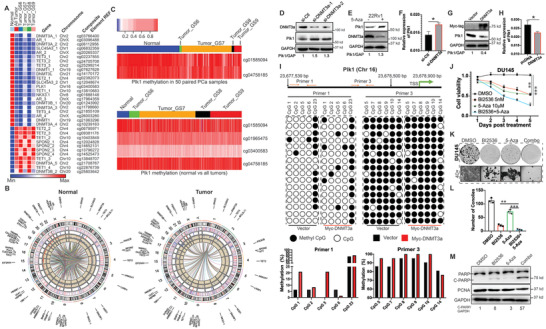
Methylation status alterations suppress transcription of Plk1. A) Alteration of methylation for genes associated with PCa progression. B) Correlations of mRNA levels and DNA methylation in normal versus tumor samples. The components starting from the outermost circle are gene names, ideogram, heatmap for mRNA expressions, heatmap for methylation levels, scatterplot for the correlations between each gene and all the methylation loci, scatterplot for the correlations between each methylation locus and all genes, and connection lines between all pairs of correlations. C) Methylation of the promotor of Plk1 in 50 paired PCa (upper panel) and total tumor samples (lower panel). D) Immunoblot of DU145 cells with depletion of DNMT3a. E) Immunoblot of 22Rv1 cells treated with or without 10 µm 5‐Aza. F) qPCR of DU145 cells transfected with siRNA against DNMT3a. G) Immunoblot and H) qPCR of HEK293T cells with overexpression of DNMT3a. I) Bisulfite Analysis of at the Plk1 promoter from cells HEK293T cells and those transfected with DNMT3a. Lollipop graphs show the methyl status of each CpG (columns) and clones (rows) in the Plk1 promoter split into 2 regions for sequencing (Primer 1‐left and Primer 2‐right). Histograms show overall methylation at each CpG site comparing vector versus DNMT3a. J) DU145 (3 × 10^3^) cells were seeded onto 96‐well plates overnight, treated with BI2536 (5 nm), 5‐Aza (10 µm), or both, and used for MTT assays. K) DU145 (1 × 10^3^) cells were seeded onto 6‐well plates, treated with BI2536 (2.5 nm), 5‐Aza (5 µm), or both, and cultured for two weeks with a medium change every three days. L) Statistic analysis of colony formation in (K). n = 3. M) Immunoblot of cells treated with 5 nm BI2536 and/or 30 µm 5‐Aza for two days. qRT‐PCR and colony formation were analyzed by unpaired Student *t*‐test. Cell viability assay was analyzed by Two‐way ANOVA test.*p<0.05, **p<0.01, ***p<0.001.

The study of DNA methylation landscape of advanced PCa indicated the important regulatory role of DNA methylation of genes including TET and DNMT in metastatic PCa.^[^ ^15^ ^]^ To further dissect how aberrations of gene methylation affect PCa progression, we downloaded 553 primary PCa patients’ data, including methylation microarray dataset and RNA sequencing profiles from TCGA, followed by analysis with R language. Total 42 763 out of 485 577 methylation probe sets and 4879 genes expression are related to PCa progression. Total of 792 hypermethylated (downregulated) genes and 4087 hypomethylated (upregulated) genes were identified. Further correlation analysis between methylation and mRNA illustrated that DNA methylation levels of AR, TET family, DNMT family, SPON2, and SLC45A3 were intimately associated with PCa progression by regulating expressions of 4879 genes, including EZH2, Plk1, and Myc, all of which have well‐documented roles in PCa. In agreement, we observed that the level of Plk1 tends to be higher in the GS9 mCRPC samples in comparison to those of other samples with GS<8. Methylation of AR, TET family, DNMT family, SPON2, and SLC45A3 apparently correlated to Plk1 gene expression in mCRPC samples (Figure S2, Supporting Information). In general, hypomethylation of DNMTs and TET3, and hypermethylation of TET1, TET2, and SPON2 were observed in tumors with increasing GS (**Figure** **3**A). Furthermore, the correlation between the methylation of DNMTs, TETs, and SPON2 genes and global gene transcription was dynamically changed in tumors with different GS, indicating that expression of specific methylation‐regulated genes contributes to PCa progression by regulating gene transcription (Figure 3B and Figure S3, Supporting Information). For example, in comparison with normal samples, hypomethylation of SLC45A3 locus significantly correlates with most of the genes in PCa tumor samples, implying its role in PCa progression (Figure 3B).

### 2.7 DNMT3a Suppresses Plk1 Transcription

In cancer, global DNA methylation patterns are altered and accompanied by the hypermethylation of CpG islands and the hypomethylation of non‐CpG islands at promoters of specific genes.^[^ ^16^ ^]^ This alteration is largely dependent on the action of de novo DNMTs during early tumor progression. Inhibitors for Plk1 are currently being tested in multiple clinical trials. It has been documented that the clinical success of microtubule‐targeting agents in PCa is attributed not solely to the induction of mitotic catastrophe in cancer cells, but also to non‐mitotic effects such as targeting intracellular trafficking on microtubules. This raises the question of whether the non‐mitotic functions of Plk1 are regulated by DNA methylation in PCa. To this end, we compared methylation of promoters of Plk1 in normal and tumor samples from TCGA. As illustrated, hypomethylation of promoters of Plk1 was observed in both paired and non‐paired tumors in comparison with normal tissues. Moreover, this hypomethylation was generally accompanied by patients with higher GS indicating a potential higher level of Plk1 in advanced PCa (Figure 3C). In agreement, this finding is in agreement with the observations in Figure 2C.

In mammals, de novo DNA methyltransferase DNMT3a contributes to the establishment of DNA methylation pattern (18). Basically, hypermethylation of a promoter region suppresses gene transcription. Therefore, we tested the hypothesis that DNMT3a increases de novo methylation of the Plk1 promoter region and represses Plk1 transcription in PCa. To this end, we depleted DNMT3a level in DU145 cells and measured the level of Plk1. As shown in Figure 3D, depletion of DNMT3a elevated the level of Plk1. We then used 5‐Aza, an inhibitor of DNMTs, to treat PCa cells resistant to enzalutamide, another advanced PCa. 5‐Aza clearly elevated the level of Plk1 in advanced (enzalutamide‐resistant) line: 22Rv1 (Figure 3E). The elevation of Plk1 is induced by knockdown of DNMT3a due to the augment of its transcription (Figure 3F). To validate these findings, we transfected DNMT3a into HEK293T cells and measured the level of Plk1. As shown in Figure 3G, transfection of DNMT3a dramatically reduced the level of Plk1, suggesting that DNMT3a is a negative regulator of Plk1. To determine whether the alteration of the protein level of Plk1 is due to transcription or post‐transcription, we next evaluated the mRNA levels of Plk1 in cells transfected with DNMT3a construct. As indicated in Figure 3H, the expression of DNMT3a reduced the mRNA level of Plk1. To directly test whether DNMT3a induces hypermethylation of the Plk1 promoter, we performed bisulfite sequencing of the Plk1 promoter of cells expressing DNMT3A. Expression of DNMT3a resulted in increased levels of methylation of the Plk1 promoter (Figure 3I). The finding that DNMT3a antagonizes Plk1 provides a rationale to combine inhibitors of DNMT3a and Plk1 to treat PCa. Accordingly, PCa cells were treated with BI2536 and/or 5‐Aza. As shown in Figure 3J, monotherapy inhibited cell growth, but the inhibitory effect was much more significant upon dual treatment. Treatment with the combination of BI2536 and 5‐Aza also significantly inhibited colony formation (Figure 3K,L). Similarly, BI2536 plus 5‐Aza treatment significantly promoted cell death (Figure 3M). To elucidate the effects of combination treatment on benign cells, we treated immortalized prostate epithelial cells, RWPE‐1 with GSK461364A and/or 5‐Aza. We observed less sensitivity to monotherapy of GSK461364A and 5‐Aza in RWPE‐1 cells than that in DU145 cells (Figure S4, Supporting Information). Moreover, the combination of GSK461364A and 5‐Aza showed more repression on cell growth of DU145 cells than that of RWPE‐1 cells. The observation that tumor cells rather than benign cells are more sensitive to mono‐ and dual treatments suggests the good tolerance of the combination of Plk1 inhibition and 5‐Aza in PCa.

5‐Aza is incorporated into the genome of proliferating cells, where it inhibits DNA methyltransferases (DNMTs), leading to a reduction of mdC. To determine whether the observed synergic effect is due to inhibition of DNMT3a, we then depleted DNMT1, DNMT3a, and DNMT3b, followed by treatment with a more specific Plk1 inhibitor GSK461364A for 48 h and measurement of cell viability. As demonstrated in Figure S5, Supporting Information, knockdown of DNMT1 and DNMT3a rather than DNMT3b showed synergic effects in cell viability when in combination with inhibition of Plk1. The effects were more significant in cells with depletion of DNMT3a than that of DNMT1. Taken together, we concluded that DNMT3a downregulates Plk1 by increasing methylation of the Plk1 promoter and that a combination of DNMT3a and Plk1 inhibitors represses advanced PCa.

### 2.8 DNMT3a Inhibits Autophagy in a Plk1‐Dependent Manner

The critical roles of Plk1 in multiple stages of mitosis have long been appreciated.^[^ ^17^ ^]^ Beyond its mitotic effects, non‐mitotic functions of Plk1, such as DNA replication^[^ ^18^ ^]^ and mTOR signaling,^[^ ^19^ ^]^ exerted important roles in human cancer. Our bioinformatics analysis described in Figure 2 showed that both DNMT3a and Plk1 are involved in autophagy, a physiological catabolic process for cell survival by which cells clean out damaged organelles and recycle nutrients when homeostasis is maintained.

**Figure 4:**
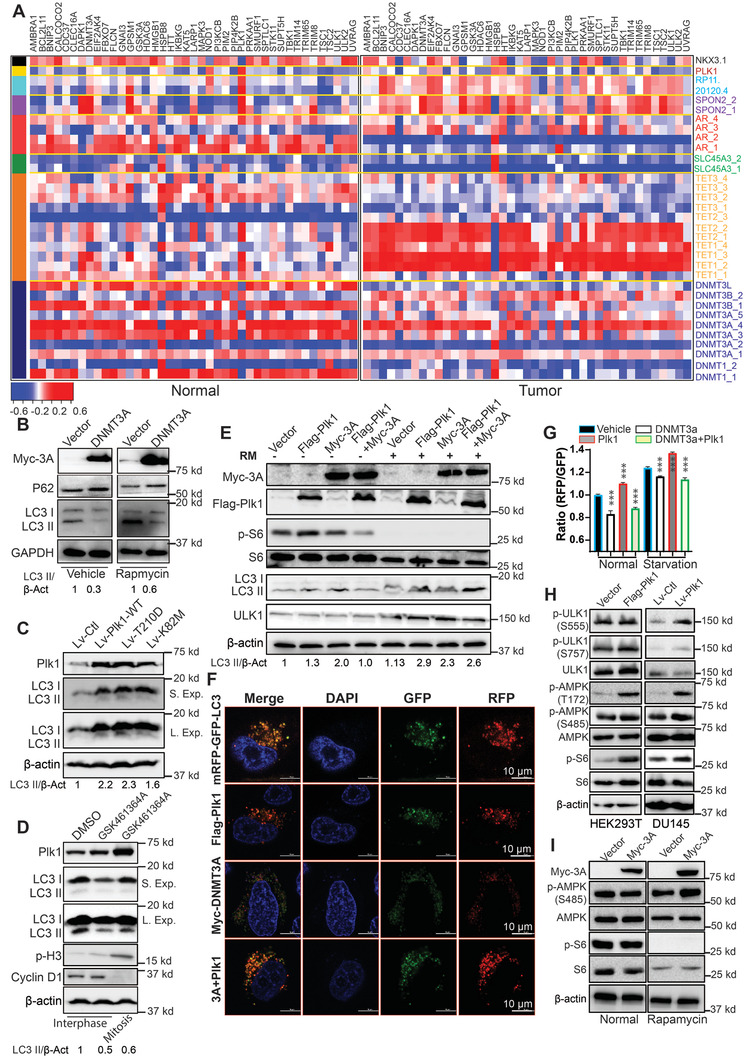
DNMT3a inhibits autophagy. A) Heatmap to compare the correlation between mRNA and DNA methylation of autophagy‐associated genes in normal and tumor specimens. B) HEK293T cells were transfected with DNMT3a and treated with 200 nm rapamycin overnight. C) DU145 cells were infected with lentivirus expressing various forms of Plk1 (WT, T210D, or K82M), subjected to starvation for 30 min, and harvested for immunoblotting (IB). D) Randomly growing DU145 cells (left two lanes) were treated with GSK461364A, and harvested. To enrich cells at mitosis (the right lane), cells were treated with nocodazole in the presence of GSK461364A, followed by shake‐off to collect floating cells. E) HeLa cells expressing DNMT3a, Plk1, or both were treated with 200 nm rapamycin overnight. F) HeLa cells were co‐transfected with DNMT3a, Plk1, and mRFP‐GFP‐LC3 reporter and subjected to IF to determine LC3 puncta formation. G) HeLa cells expressing DNMT3a, Plk1, or both were starved overnight and subjected to flow cytometry. n = 3 for each groups. Flow cytometry data were analyzed by unpaired Student *t*‐test. H) Immunoblot of HEK293T cells transfected with Plk1 and DU145 cells infected with lentivirus expressing Plk1. I) HEK293T cells were transfected with DNMT3a, starved for 2 h, and harvested for IB. **p<0.01, ***p<0.001.

Herein, we sought to investigate whether the autophagy pathway, identified by XDeath, was regulated by aberration of DNA methylation in PCa. To this end, we compared the expression profile of autophagy‐related genes that are regulated by methylation alteration in normal and tumor samples. As shown in **Figure** 4A and Figure S6, Supporting Information, autophagy‐related genes, especially those regulated by DNMT family and TET family, were significantly changed during disease progression, suggesting activation of the autophagy pathway in later stage PCa. To further dissect how the newly identified Plk1/DNMT3a axis contributes to autophagy, we transfected cells with DNMT3a plasmid, followed by inhibition of mTORC1 with rapamycin. As demonstrated in Figure 4B, expression of DNMT3a dramatically reduced the level of LC3 II, but increased the level of P62, suggesting that DNMT3a inhibits autophagy. In contrast, expression of Plk1, in particular, the constitutively active Plk1‐T210D, elevated the level of LC3 II, indicating that Plk1 stimulates autophagy (Figure 4C). In agreement, inhibition of Plk1 by administrating GSK461364A significantly reduced the level of LC3 II, indicating inhibition of autophagy (Figure 4D). The autophagy analyses described above were performed in non‐synchronized cells. However, we could not rule out that Plk1 may regulate autophagy in mitotic cells as well. Accordingly, we also analyzed LC3 II during mitosis with mitotic cells being collected by shake‐off. Autophagy, monitored by LC3‐II levels, was not reduced in mitotic cells as compared with control cells remained. Thus, we conclude that mitotic cells display low autophagy, suggesting that Plk1 may promote autophagy primarily in non‐mitotic cells (Figure 4D). To further confirm the roles of DNMT3a and Plk1 in autophagy, we then treated transfected cells with rapamycin to induce autophagy. We showed that overexpression of DNMT3a antagonized rapamycin‐induced autophagy (Figure 4E). In contrast, overexpression of Plk1 robustly promoted rapamycin‐induced autophagy, consistent with a previous report^[^ ^20^ ^]^ (Figure 4E). We also examined the role of Plk1 in autophagy by immunofluorescence and flow cytometry to follow the formation of LC3‐positive puncta. We observed that Plk1 significantly enhanced starvation‐induced formation of LC3‐positive puncta, which can be attenuated by DNMT3a coexpression (Figure 4F,G). To further dissect mechanisms contributing to autophagy, we manipulated Plk1 and DNMT3a in cells and showed that expression of Plk1 activated ULK1, as indicated by the increase of phosphorylation at S555. However, no significant changes were observed for phosphorylation of ULK1 at S757. In agreement, mTORC1 pathway activation demonstrated by p‐S6 upon Plk1 expression suggests that Plk1‐induced autophagy is independent of mTORC1 pathway. Furthermore, expression of Plk1 activated AMPK by inducing p‐AMPK (T172) rather than p‐AMPK (S485). These results suggest that activation of ULK1 is dependent on Plk1 (Figure 4H). Correspondingly, inhibitory phosphorylation of AMPK at S485 was elevated in cells expressing Myc‐DNMT3a upon rapamycin treatment (Figure 4I). These results suggest that DNMT3a suppresses autophagy. Moreover, no changes in mRNA level of UK1 were observed after overexpression of DNMT3a (data not shown). Taken together, DNMT3a‐mediated inhibition of autophagy is Plk1‐dependent.

### 2.9 Plk1‐Associated Activity Results in DNMT3a Degradation in Mitosis

**Figure 5:**
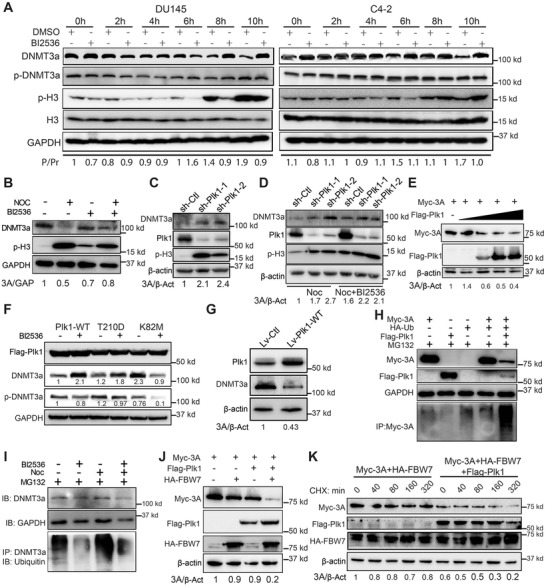
Cell cycle‐dependent expression of DNMT3a is regulated by Plk1‐associated kinase activity. A) DU145 and C4‐2 cells were synchronized by double thymidine block (DTB, treatment with thymidine for 16 h, release for 8 h, and a second thymidine treatment for 16 h), released into fresh medium ± 100 nm BI2536 for various times and harvested for IB and quantification of p‐DNMT3a/DNMT3a. P, phosphorylated DNMT3A; Pr, total DNMT3a. B) Immunoblot of HeLa cells treated with 100 ng mL^−1^ nocodazole ± 50 nm BI2536 for 12 h. C) Immunoblot of DU145 cells depleted of Plk1 with shRNA. D) DU145 cells were depleted of Plk1 with shRNA, and treated with 50 ng mL^−1^ nocodazole ± 50 nm BI2536 overnight. E) HEK293T cells were co‐transfected with Myc‐DNMT3a and increasing amounts of Flag‐Plk1. F) HEK293T cells were transfected with Plk1 (WT, T210D, or K82M) and treated with 100 nm BI2536 for 12 h. G) Immunoblot of 22Rv1 cells infected with lentivirus expressing Plk1. H) HeLa cells transfected with Myc‐DNMT3a, HA‐Ub, and Flag‐Plk1 were treated with 10 µm MG132 for 16 h and harvested for anti‐Myc immunoprecipitation (IP), followed by anti‐ubiquitin IB. I) HeLa cells were synchronized with nocodazole, incubated with 50 nm BI2536 for 24 h, and harvested for anti‐DNMT3a IP, followed by anti‐ubiquitin IB. J) HEK293T cells transfected with Myc‐DNMT3a, Flag‐Plk1, and HA‐FBW7 were harvested for IB. K) HEK293T cells transfected with Myc‐DNMT3a, HA‐FBW7, and/or Flag‐Plk1 were treated with 25 µg mL^−1^ cycloheximide and harvested at various times for IB.

Besides the non‐mitotic role, Plk1 is a key regulator of cell‐cycle progression and a key target for the treatment of human cancer. The expression of Plk1 starts at the S phase with low enzymatic activity and peaks at G2/M.^[^ ^17^ ^]^ Considering the fundamental role of Plk1 in cell cycle, it's feasible to ask whether the levels of DNMT3a are cell‐cycle regulated, as Plk1 is a mitotic kinase. As indicated in **Figure** **5**A, the level of total DNMT3a significantly decreased in mitosis, and the presence of Plk1 inhibitor BI2536 prevented the decrease. Because the total level of DNMT3a is cell‐cycle regulated, we followed the pS390/3‐DNMT3a/DNMT3a ratio. Quantification indicated that phosphorylation of DNMT3a did increase as cells entered mitosis and that BI2536 inhibited this increase (Figure 5A). Next, we arrested cells in mitosis with nocodazole and examined the level of DNMT3a under various conditions. As expected, nocodazole treatment significantly reduced the level of DNMT3a, but the addition of BI2536 partially reversed this effect (Figure 5B). Furthermore, depletion of Plk1 elevated the level of DNMT3a (Figure 5C). This elevation was remained even in cells arrested at mitosis by nocodazole and cannot be attenuated by BI2536 upon depletion of Plk1 (Figure 5D). In addition, the expression of Plk1 induced the reduction of Myc‐DNMT3a in a dose‐dependent manner (Figure 5E). To further examine whether the reduced level of DNMT3a is due to Plk1 activity, we transfected HEK293T cells with different forms of Plk1, wild type (WT), constitutively activated T210D mutant (TD), and kinase defective K82M mutant (KM), and treated the cells with BI2536, followed by IB to quantify the levels of DNMT3a and p‐DNMT3a. Overexpression of Plk1‐TD led to the highest level of p‐DNMT3a, whereas cells expressing Plk1‐KM had a much lower level of p‐DNMT3a. Finally, the combination of BI2536 and Plk1‐KM expression almost completely abolished the phosphorylation of DNMT3a (Figure 5F). In agreement, the total level of DNMT3a in cells expressing Plk1‐WT and ‐TD were much lower than that in cells expressing Plk1‐KM (compare lanes 1, 3, 5 of Figure 5F). Next, we want to ask whether Plk1‐induced reduction of DNMT3a is observed in PCa cells. As shown in Figure 5G, overexpression of Plk1 reduced the level of DNMT3a in 22Rv1 cell line. The collective results indicate that Plk1‐associated kinase activity contributes to protein degradation of DNMT3a in mitosis.

### 2.10 Plk1‐Associated Activity Promotes Proteasome‐Dependent DNMT3a Degradation

To distinguish whether the decrease of DNMT3a in mitosis is due to reduced transcription or to altered protein modification that contributes to destabilization, we first compared mRNA levels of DNMT3a in DU145 cells that had been treated with or without BI2536 for 0 and 10 h after the DTB. We found that there was no significant difference among the different groups (Figure S7, Supporting Information), suggesting that transcriptional alteration does not contribute to the change in the level of DNMT3a protein. We then evaluated the role of protein modification by ubiquitination assay. As shown in Figure 5H, a much higher level of polyubiquitination of DNMT3a was detected upon co‐expression of Plk1. Furthermore, nocodazole treatment significantly increased ubiquitination of DNMT3a, and this was attenuated by BI2536 (Figure 5I). Next, we screened for E3 ligase that contributed to the degradation of DNMT3a. As showed in Figure 5J, expression of FBW7 reduced the level of DNMT3a upon Plk1 overexpression, suggesting that Plk1‐induced ubiquitination of DNMT3a was FBW7 dependent. To further rule out the regulation of DNMT3a at the post‐transcriptional level, we treated cells with cycloheximide, an inhibitor of protein translation, followed by measurement of protein turnover. As indicated, overexpression of Plk1 decreased the level of Myc‐DNMT3a upon cycloheximide treatment (Figure 5K). Taken together, we conclude that Plk1‐associated kinase activity contributes to DNMT3a degradation through activation of the proteasome pathway.

### 2.11 Plk1 Directly Phosphorylates DNMT3a at S393

**Figure 6:**
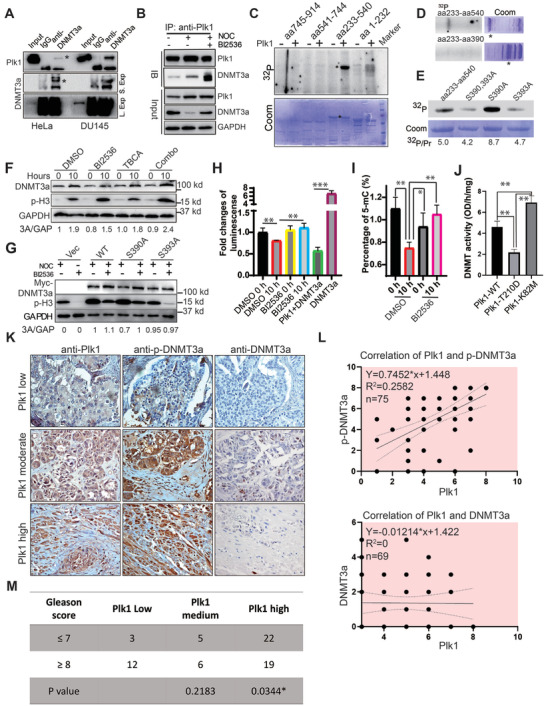
Phosphorylation of DNMT3a‐S393 results in its degradation in mitosis. A) HeLa and DU145 cells were subjected to anti‐DNMT3a IP, followed by anti‐Plk1 IB. B) DU145 cells were treated with BI2536 plus nocodazole for 10 h and subjected to anti‐Plk1 IP, followed by anti‐DNMT3a IB. C) Plk1 targets the C‐terminus of DNMT3a. Purified Plk1 was incubated with purified GST‐DNMT3a regions (aa1‐232, 233–540, 541–744, and 745–914) in the presence of [*γ*‐^32^P] ATP. The reaction mixtures were resolved by SDS‐PAGE, stained with Coomassie brilliant blue (Coom.), and detected by autoradiography. D) Plk1 was incubated with GST‐DNMT3a regions (aa233‐540 and 233–390) as in (C). E) Plk1 was incubated with recombinant DNMT3a‐aa 233–540 (WT or various mutants) as in (C). F) Cells were synchronized with the DTB, released into a medium containing BI2536 and/or TBCA for 10 h. G) Cells were transfected with different DNMT3a constructs (WT, S390A, or S393A), synchronized with nocodazole for 10 h in the absence or presence of BI2536. H) DU145 cells were synchronized with the DTB, released into a medium containing BI2536 for 10 h, and DNMT3a activity was assayed. For the samples represented by two columns at right, purified DNMT3a was incubated with or without purified Plk1 prior to measurement of activity. n = 3 for each groups. I) Samples as in (G) were subjected to global DNA methylation assay. n = 3 for each groups. J) HEK293T cells were co‐transfected with DNMT3a and Plk1 (WT, T210D, or K82M) and harvested for DNA methyltransferase assays. n = 3 for each groups. K) Immunohistochemistry staining with anti‐Plk1, anti‐p‐DNMT3a, and anti‐DNMT3a in a human PCa tumor microarray (TMA). Magnification: 40 ×. L) Correlation between Plk1 and p‐DNMT3a (n = 75) and DNMT3a (n = 69) in TMA. M) Correlation between the levels of p‐DNMT3a and PCa progression. Luciferase assay, 5‐mC level, and DNMT activity data were analyzed by unpaired Student *t*‐test. Correlations of Plk1 and DNMT3a and p‐DNMT3a were analyzed by linear regression test. *p<0.05, **p<0.01, ***p<0.001.

To understand the mechanisms by which Plk1 contributes to DNMT3a degradation, HeLa and DU145 cells were harvested for anti‐DNMT3a IP, followed by anti‐Plk1 IB. We found that DNMT3a directly binds to Plk1 (**Figure** **6**A). Next, we further explored whether the binding is cell‐cycle dependent. To this end, DU145 cells were arrested at mitosis with nocodazole ± BI2536 and harvested for anti‐Plk1 IP, followed by anti‐DNMT3a IB. We found that DNMT3a directly binds to Plk1, in particular, in mitosis (Figure 6B). Using recombinant GST‐DNMT3a fragments, we showed that a DNMT3a fragment containing amino acids (aa) 390 to 540 was phosphorylated by Plk1 (Figure 6C,D). To map the phosphorylation site(s), we individually mutated each serine/threonine within the region to alanine, and eventually identified S393 as the Plk1 phosphorylation site (Figure 6E). In a previous study, S390 and S393 were reported as CK2 phosphorylation sites. To evaluate the potential contribution of CK2‐mediated phosphorylation in DNMT3a degradation, DU145 cells were treated with 40 nm BI2536 or 10 µm TBCA, an inhibitor of CK2, upon the release from DTB and analyzed. The results indicate that both Plk1 and CK2 contribute to the degradation of DNMT3a in mitosis and that a combination treatment with BI2536 and TBCA resulted in the best reversal for mitosis‐induced DNMT3a degradation (Figure 6F). To distinguish the contribution of Plk1 versus CK2 in the phosphorylation events, DU145 cells were transfected with various DNMT3a constructs (WT, S390A, and S393A), and treated with 100 nm BI2536 for 10 h after 100 ng mL^−1^ nocodazole treatment. We found that the S390A mutant behaved like WT DNMT3a, suggesting that phosphorylation of S390 by CK2 does not affect its degradation in mitosis. In contrast, the S393A mutant was clearly stabilized in mitosis, indicating that Plk1‐ and CK2‐dependent phosphorylation of S393 results in DNMT3a degradation in mitosis (Figure 6G). Considering the fact that Plk1 but not CK2 is cell‐cycle regulated, Plk1 is likely the major kinase responsible for the mitosis‐specific degradation of DNMT3a. We also directly compared DNMT3a activities under different conditions. As expected, mitosis‐enriched cells (10 h after release from the DTB) showed reduced DNMT3a activity, which was reversed by BI2536. Pre‐incubation of purified DNMT3a with recombinant Plk1 also significantly reduced its activity (Figure 6H). In agreement, global DNA methylation was reduced in mitosis‐enriched cells, and BI2536 treatment prevented the decrease (Figure 6I). Further, co‐expression of Plk1‐T210D reduced the level of DNMT3a activity, but co‐expression of Plk1‐K82M enhanced the level of DNMT3a activity (Figure 6J). To determine whether this novel phosphorylation event is of clinical significance, we performed immunohistochemistry staining with anti‐Plk1 and anti‐p‐DNMT3a antibodies in a human PCa tissue array. We did observe a correlation between the level of DNMT3a phosphorylation and the level of Plk1, supporting the notion that Plk1 phosphorylates DNMT3a in patients as well as in vitro (Figure 6K,L). Even in some cases, we could observe negative correlation between Plk1 and DNMT3a. However, we did not observe the statistical significance of the correlation between these two proteins in human PCa TMA (Figure 6K,L). In addition, patients with a lower level of p‐DNMT3a significantly correlated with poor prognosis (GS≥8) (Figure 6M). In sum, we conclude that Plk1 phosphorylation of DNMT3a at S393 is a major regulatory mechanism that inhibits its activity.

### 2.12 Plk1 Inhibition Enhances the Efficacy of 5‐Aza in PCa Xenograft Tumors

The mutual regulation between DNMT3a and Plk1 demonstrated above provides a strong rational to test whether Plk1 inhibition and 5‐Aza can inhibit advanced PCa, such as enzalutamide‐resistant CRPC synergistically. To this end, we treated mice carrying 22Rv1 xenograft tumors with BI2536, 5‐Aza, or both for 6 weeks. As shown in Figure S8A, Supporting Information, BI2536 or 5‐Aza alone slightly decreased the tumor volume, whereas the combination of both drugs synergistically decreased tumor volume, indicating that BI2536 plus 5‐Aza is effective to treat enzalutamide‐ resistant CRPC. Although both wet weight and size of the tumors were reduced with monotherapy of BI2536 or 5‐Aza, the effect was more significant with combined treatment (Figure S8B,S8C, Supporting Information). Hematoxylin and Eosin (H&E) staining of tumors from the combined‐treated mice showed more necrosis and increased numbers of apoptotic bodies in comparison with the tumors from monotherapy‐treated mice. For the untreated group, we detected more mitotic cells, suggesting that cell division was active (Figure S8D, Supporting Information). To determine whether BI2536 or 5‐Aza alone or in combination represses tumor proliferation and promotes apoptosis, tumor samples were analyzed by immunofluorescence staining for Ki67 and cleaved caspase‐3. As shown in Figure S8E,S8F, Supporting Information, 5‐Aza or BI2536 inhibited Ki67 expression and increased the level of cleaved caspase‐3, but the combination treatment further decreased the level of Ki67 and increased the level of cleaved caspase‐3.

**Figure 7:**
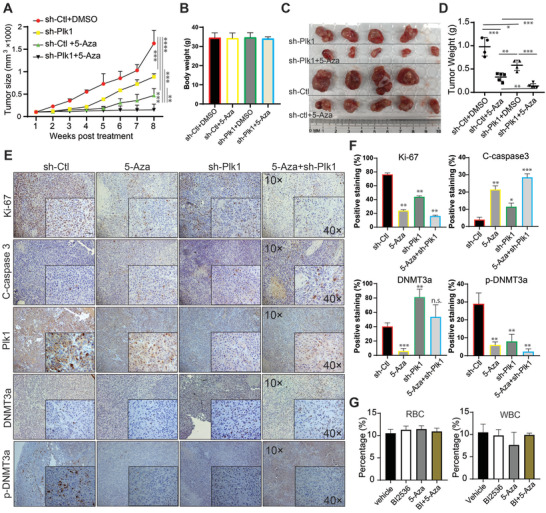
Depletion of Plk1 and inhibition of DNMT3a suppress DU145‐derived xenograft tumor growth synergistically. A) Nude mice were inoculated with DU145 cells depleted of Plk1 (2.5 × 10^5^) with lentivirus and treated with 5‐Aza (3.3 mg kg^−1^, intraperitoneal injection), followed by measurement of tumor size every week (means ± SE). Tumor growth for different groups was analyzed by Two‐way ANOVA test. B) Body weight was measured at the end of the study. C) Tumor images at the end of the study. D) Wet weights of tumors. E) IHC staining for Ki67, cleaved caspase 3, Plk1, DNMT3a, and p‐DNMT3a in xenograft tumors. F) Quantification of (E). G) Quantification of RBC and WBC in mice received treatments. n = 4 for sh‐ctl and sh‐Plk1 groups, n = 5 for sh‐ctl+5‐Aza and sh‐Plk1+5‐Aza groups. Tumor mass data were analyzed by unpaired Student *t*‐test. *p<0.05, **p<0.01, ***p<0.001.

To rule out the potential off‐target effects of BI2536 on tumor suppression in vivo, a more stable genetic approach (Plk1 shRNA) was used. DU145 cells were depleted of Plk1 with shRNA and inoculated into nude mice. Upon tumor formation, the mice were treated with 5‐Aza for 8 weeks. As shown in **Figure** **7**A, Plk1 depletion or 5‐Aza alone slightly decreased tumor growth, but Plk1 depletion plus 5‐Aza resulted in an almost complete block of tumor growth. Of note, the combination treatment did not affect animal weight, suggesting that possible side effects for co‐targeting Plk1 and DNMT3A are tolerable (Figure 7B). Both wet weight and size of the tumors were reduced with monotherapy of 5‐Aza, but the effect was much more significant with 5‐Aza plus Plk1 depletion (Figure 7C,D). To determine whether 5‐Aza alone or in combination with Plk1 depletion represses tumor cell proliferation and promotes apoptosis, tumor samples were analyzed for Ki67 and cleaved caspase‐3 staining. As shown in Figure 7E,F, Plk1 depletion or 5‐Aza alone inhibited Ki67 expression and promoted caspase‐3 activation, but the combination treatment further decreased the level of Ki67 and significantly increased the level of cleaved caspase‐3. In addition, depletion of Plk1 elevated the level of DNMT3a but reduced the level of p‐DNTMT3a in xenograft tumors (Figure 7E,F). To examine treatment‐associated toxicity within each group, we assessed blood cells and performed histopathologic examination of vital organs. Overall, no significant pathological changes were detected in any group. No obvious changes for red blood cells and white blood cells were observed in any group (Figure 7G). We conclude that both 5‐Aza/BI2536 alone and combination treatment are well tolerated. These findings in the xenograft mouse model render the potential to improve treatment efficacy for PCa patients with this combinational strategy because mono treatment with 5‐Aza upregulates Plk1 expression which provides targets for Plk1 inhibitors. Administration of Plk1 inhibitor could reverse the side effects induced by DNMT3a inhibition. In agreement with our findings, many kinds of inhibitors of DNMTs and Plk1 applied in clinical trials showed good safety and tolerance providing potentials for application in patients.

## 3 Discussion

Cell death is a physiological process critical for the normal development and function of multicellular organisms.^[^ ^21^ ^]^ To date, twelve different cell‐death modalities have been recognized by the NCCD, with apoptosis (type I), autophagy (type II), and necrosis (type III)^[^ ^22^ ^]^ as three main types of cell death. Increased cell survival with pathophysiological consequences that lead to resistance to cell death is an early hallmark of developing cancer. Induction of cell death is currently the primary goal of therapy for most cancers, including PCa.^[^ ^23^ ^]^ Further, it has been described that deregulation of various cell‐death pathways is linked to the pathogenesis of PCa progression.^[^ ^24^ ^]^

With the goal to identify novel targets/pathways involved in PCa progression, we constructed a systems biology model, XDeath, to gain insights in pathway crosstalk to dissect twelve cell‐death modes. To our knowledge, this is the first construction of a bioinformatics model to study PCa progression with dynamic pathway alterations. With this method, we will be guided macroscopically to identify novel targets for PCa treatment, especially for advanced PCa. Our bioinformatics XDeath model provides strong evidence that the aforementioned pathways may be regulated by cytokines and growth factors in PCa. Major signaling pathways regulating cell growth and death, such as JAG2, BRCA2, CDK10, HRAS, BCL2, EZH2, DNMT, and MYC have been identified (Figure 1C). Plk1, which has a well‐documented role in cell proliferation and is a target for PCa,^[^ ^10^ ^]^ was also identified. Our results are also in agreement with reports in which proteomics data analysis showed that the dysregulation of cell‐cycle function is the most possible reason for PCa progression. Further, genetic modifications of BRCA2, ATM, EZH2, and CTNNB1 have been associated with poor PCa prognosis, validating our novel computational approach.

Cell death is an interacting consequence of multiple signaling events, as there are extensive crosstalk among various cell‐death mechanisms.^[^ ^25^ ^]^ In this paper, cell‐death modes have been investigated in a systematic manner during PCa progression. By identifying dysregulated cell‐death mechanisms, the XDeath model offers the possibility of developing novel treatments for late‐stage PCa by directly activating cell‐death machinery. Based on our computational prediction, we recommend several novel therapies that co‐target pathways with crosstalk in late PCa (Table S10, Supporting Information). The top 3 combinations include HSP90 and AR, IGF1R and CDK10, and Plk1 and DNMT3a. We validated the Plk1/DNMT3a axis in great detail.

Plk1, the prototypical member of the polo‐like family of serine/threonine kinases, is a pivotal regulator of mitosis and cytokinesis in eukaryotes. Plk1 is overexpressed in many cancers, including PCa,^[^ ^10^ ^]^ and its depletion leads to PCa cell death.^[^ ^26^ ^]^ DNMT3a, whose expression level is elevated in PCa, is a valid target as well.^[^ ^27^ ^]^ However, whether and how Plk1 interacts with DNMT3a to contribute to PCa progression is unknown. The unbiased approach, XDeath, suggests that the interaction between Plk1 and DNMT3a might contribute to PCa progression via regulation of autophagy (Figure 2).

To validate our computation‐based findings, we performed a series of experiments showing that DNMT3a functions as a negative regulator of Plk1 expression via hypermethylation of the Plk1 promoter in interphase cells, suggesting a negative feedback loop between these two proteins. In other words, when we use 5‐Aza to inhibit DNMT3a, Plk1 transcription is increased due to hypomethylation of its promoter. Further, we also demonstrated that Plk1 directly phosphorylates DNMT3a and that Plk1 phosphorylation of DNMT3a at S393 leads to its protein degradation and reduced DNMT activity. On the other hand, when we used BI2536 to inhibit Plk1, DNMT3a was stabilized due to dephosphorylation at S393, and elevated DNMT3a further inhibited Plk1 expression. Elevated Plk1 further inhibited DNMT3a via phosphorylation at S393 in mitosis in accord with its mitotic role during cell cycle. Such a novel mutual negative regulation mechanism between Plk1 and DNMT3a provides a strong rationale to combine inhibition of Plk1 and DNMT3a for treatment of PCa (Figure S9, Supporting Information).

Autophagy is an adaptive biological process involved in various cellular homeostasis by facilitating nutrition utilization and removing toxins. Given the roles of autophagy in tumor pathogenesis, inhibition of autophagy raises as an emerging regimen of research interest. Autophagy inhibition is now being explored in various clinical trials for patients with refractory malignancies including PCa.^[^ ^28^ ^]^ Many autophagy inhibitors being investigated in PCa research including chloroquine, metformin in clinical trials.^[^ ^29^ ^]^ In the present study, bioinformatics analysis revealed autophagy as a common death mode shared by Plk1 and DNMT3a (Figure 2H). In addition to the mitotic role of Plk1 as a cell‐cycle related kinase, the regulation of its non‐mitotic signaling plays important roles in cancer as well. As illustrated, Plk1 promotes autophagy whereas DNMT3a expression inhibits this process independent of its mitotic effects (Figure 4). So, when Plk1 is inhibited by BI2536, autophagy will be repressed. In contrast, when DNMT3a is inhibited by 5‐Aza, autophagy will be activated. At the same time, Plk1 transcription will be elevated due to hypomethylation of its promoter, resulting in further activation of autophagy. Again, such a negative feedback loop mechanism between Plk1 and DNMT3a also supports the use of a combination of BI2536 and 5‐Aza to treat late‐stage PCa (Figure S9, Supporting Information). Our in vivo data based on xenograft models clearly supports this notion (Figure 7). Given the metastatic PCa cell line, DU145 was applied in vitro and in vivo and significant efficacy of the combination of Plk1i plus 5‐Aza was observed, it's rational to expect acceptable efficacy of the combinational strategy for spinal cord metastases. These findings suggest that a future clinical trial combining both Plk1 and DNMT3a inhibitors would be worthwhile.

## 4 Experimental Section

##### Patient Samples

The total PCa cohorts consisted of 679 patients with 729 transcriptome profiles, including: 1) 497 PCa patients from TCGA, in which 55 patients have the associated adjacent normal samples, and 2) GSE21034 dataset included 177 PCa patients as an independent validation dataset (Table S1, Supporting Information). All samples were derived from fresh‐frozen tissue specimens before any treatment. All transcriptome RNA sequencing datasets were collected from the database Gene Expression Omnibus (GEO, https://www.ncbi.nlm.nih.gov/geo/) and the Cancer Genome Atlas (TCGA, https://www.cancer.gov/about‐nci/organization/ccg/research/structural‐genomics/tcga). GSE21034 was tested by Affymetrix Human Exon 1.0 ST Array, and TCGA RNA sequencing (RNA‐Seq) was obtained with platform Illumina HiSeq 2000. TCGA‐PRAD and GSE21034 were used as training tests. To investigate genes involved in PCa progression, patients in both TCGA and GEO cohorts were classified into five groups: GS< = 5 (adjacent normal), GS = 6 (tumor good), GS = 7 (tumor intermediate), GS = 8 (tumor aggressive), and GS> = 9 (tumor most aggressive) (Tables S3 and S4, Supporting Information).

##### XDeath for Modeling of Signaling Crosstalk in Disease Progression Networks

XDeath was a dynamic data‐driven network‐based model by graphical regression integration with density‐clustering to infer the crosstalk pathways of cell death during multiple space variations, such as GS, to seek the optimal cancer targets. Details can be seen in Supporting Information.

##### Cell‐Death Pathway Profiling and Network Ontology

A Cell‐Death Engine called Crosstalk Death‐DB was created (https://pcm2019.shinyapps.io/LijunLab_Pathway_Enrichment/). Based on definitions of 12 cell‐death modes described in 2018,^[^ ^30^ ^]^ literature in PubMed was reviewed and organized 149 hallmark genes of cell death, 116 cell‐death pathways with 2275 nodes (genes), and 21 488 interactions between genes across 12 cell‐death modes. XDeath built the crosstalk of global human pathways, including 116 pathways across 12 cell‐death modes during variations in GS. This approach could dissect molecular mechanisms associated with PCa progression as illustrated in Figure 1A by six steps (See Supporting Information for details).

##### Plk1 Kinase Assay

In vitro kinase assays were performed with TBMD buffer (50 mm Tris [pH 7.5], 10 mm MgCl_2_, 5 mm dithiothreitol, 2 mm EGTA, 0.5 mm sodium vanadate, and 20 mm p‐nitrophenyl phosphate) supplemented with 125 µm ATP and 10 µCi of [*γ*‐^32^P] ATP at 30 °C for 30 min in the presence of purified GST‐DNMT3a and Plk1. After the reaction, mixtures were resolved by SDS‐PAGE, the gels were stained with Coomassie brilliant blue, dried, and subjected to autoradiography.

##### DU145‐Derived Mouse Xenograft Model

All animal experiments described in this study were approved by the University of Kentucky Animal Care and Use Committee. DU145 cells were inoculated subcutaneously into nude mice (Harlan Laboratories). Two weeks later, animals were randomized into treatment and control groups. 5‐Aza was injected into the abdominal cavity twice a week at the dose of 3.3 mg kg^−1^. Tumor volumes were calculated from the formula V = L × W^2^/2 (where V is volume [cubic millimeters], L is length [millimeters], and W is width [millimeters]).

##### Bisulfite Sequencing, Pyrosequencing, and Analysis

To analyze the DNA methylation status of Plk1 promoter, freshly isolated genomic DNA (Qiagen DNeasy Blood and Tissue Kit) was digested with HindIII (NEB R0104). 500 ng of digested DNA was used in bisulfite conversion using the EZ DNA Methylation Kit (Zymo Research D5001) with conversion at 50 °C for 60 min followed by 30 s at 95 °C repeating for 16 cycles. DNA was eluted in 12ul and 2 ul were used in PCRs. Primers for PCR were designed with the help of ZymoResearch Bisulfite Primer Seeker (http://bpsbackup.zymoresearch.com) using the Plk1 promoter region, two primer pairs were created to cover the region Primer Pair 1 (Forward: 5’‐ GGGGTTTTGGTATTGTGTTTTTTTAATTTTAGGATG‐3’, Reverse: 5’‐ CTACRCAACAACAACCTTTAAACCC‐3’) and Primer Pair 3 (Forward: 5’‐ ATAAATTTTAATTTTTATGAGTTTTTTTTTTAAATG‐3’, Reverse: 5’‐ ATATATCTTACRAAATTTTATTTAACCTCCCCCC‐3’). PCR conditions using ZymoTaq PreMix were: 1) 95 °C 10 min; 2) 95 °C for 30 s; 3) 55 °C for 40 s; 4) 72 °C for 45 s; 5) Repeat 2–4 for 40 total cycles; 6) 72 °C for 7 min. PCRs were run on 1.5% agarose gel, bands were cut, and purified (Qiagen Gel Extraction). Purified bands were blunt‐end modified (End‐It DNA End‐Repair Kit‐ EpiCentre) and subcloned into pUC19 (predigested with HincII). At least 20 clones from each group were pyrosequenced (University of Chicago Comprehensive Cancer Center DNA Sequencing and Genotyping Facility). Sequences were aligned to the genomic (non‐converted sequence) using BiQ analyzer. This program aligned sequences, determined methylated Cytosine residues (in CpG context via conversion or not), and calculated methylation percent from samples at CpG sites.

##### Statistical Analysis

qRT‐PCR, cell viability assay, colony formation, flow cytometry, IF staining, IHC staining, luciferase assay, 5‐mC level, DNMT activity, and tumor mass data were analyzed by unpaired Student *t*‐test. Correlations of Plk1 and DNMT3a and p‐DNMT3a were analyzed by linear regression test. Tumor growth for different groups was analyzed by Two‐way ANOVA test. All data were presented as the mean ± SEM. Data were analyzed using the GraphPad Prism 8 software package. *P < 0.05; **P < 0.01; ***P < 0.001; ****P < 0.0001; n.s., not significant.

## Conflict of Interest

The authors declare no conflict of interest.

## Author Contributions

Z.Z. and L.C. contributed equally to this work. Z.Z. conceived the project. T.H. and L.C. developed XDeath systems and built the online platform. Z.Z., L.C., and X.L. oversaw the project, designed experiments, and interpreted data. Z.Z., L.C., and X.L. co‐wrote the manuscript. Q.Z., Y.K., and K.L. performed partial experiments. M.R and Y.N.F helped on bisulfite sequencing. R.W., J.L., Z.L., and E.L. reviewed writing. C.Y. provided reagents. D.H, C. W, and L.L helped with bioinformatics analysis.

## Supporting information

Supporting Information

Supporting Table 3.1

Supporting Table 3.2

Supporting Table 4

Supporting Table 5

Supporting Table 6.1

Supporting Table 6.2

Supporting Table 7

Supporting Table 8.1

Supporting Table 8.2

Supporting Table 8.3

Supporting Table 10

Supporting Table 11

Supporting Table 12

## Data Availability

Research data are not shared.
