# Co‐Targeting Plk1 and DNMT3a in Advanced Prostate Cancer

**DOI:** 10.1002/advs.202202572

**Published:** 2022-07-15

**Authors:** Zhuangzhuang Zhang, Lijun Cheng, Qiongsi Zhang, Yifan Kong, Daheng He, Kunyu Li, Matthew Rea, Jianlin Wang, Ruixin Wang, Jinghui Liu, Zhiguo Li, Chongli Yuan, Enze Liu, Yvonne N. Fondufe‐Mittendorf, Lang Li, Chi Wang, Xiaoqi Liu, Tao Han

*Adv. Sci*. **2021**, *8*, 2101458

DOI: 10.1002/advs.202101458

In the original published article, the name of Jianlin Wang was spelt incorrectly. The updated author byline is shown above.

