# Supplementary material for: Co‐Targeting Plk1 and DNMT3a in Advanced Prostate Cancer
Source: Adv Sci (Weinh). 2021 May 29;8(13):2101458. doi: 10.1002/advs.202101458 (PMC8261504; doi:10.1002/advs.202101458)
Supplement: Supplementary file 1 — Supporting Information [file ADVS-8-2101458-s007.pdf]

© 2021 Wiley-VCH GmbH

ADVANCED SCIENCE Open Access

Supporting Information

for *Adv. Sci.*, DOI: 10.1002/advs.202101458

Co-targeting Plk1 and DNMT3a in Advanced Prostate Cancer

Zhuangzhuang Zhang[1#], Lijun Cheng[2#], Qiongsi Zhang[1], Yifan Kong[1], Daheng He[3], Kunyu Li[1], Matthew Rea[4], Jianling Wang[1], Ruixin Wang[1], Jinghui Liu[1], Zhiguo Li[1], Chongli Yuan[5], Enze Liu[2], Yvonne N. Fondufe-Mittendorf[4], Lang Li[2] , Chi Wang[3] and Xiaoqi Liu[1,3]*

Supporting Information

Co-targeting Plk1 and DNMT3a in Advanced Prostate Cancer

Zhuangzhuang Zhang[1#], Lijun Cheng[2#], Qiongsi Zhang[1], Yifan Kong[1], Daheng He[3], Kunyu Li[1], Matthew Rea[4], Jianling Wang[1], Ruixin Wang[1], Jinghui Liu[1], Zhiguo Li[1], Chongli Yuan[5], Enze Liu[2], Yvonne N. Fondufe-Mittendorf[4], Lang Li[2] , Chi Wang[3] and Xiaoqi Liu[1,3*]

[1]Department of Toxicology and Cancer Biology, University of Kentucky, Lexington, KY, 40536, USA

[2]Department of Biomedical Informatics, The Ohio State University, Columbus, OH, 43210, USA

[3]Markey Cancer Center, University of Kentucky, Lexington, KY, 40536, USA

[4]Department of Molecular and Cellular Biochemistry, University of Kentucky, Lexington, KY 40536, USA

[5]School of Chemical Engineering, Purdue University, West Lafayette, IN 47907, USA

Keywords: Plk1, DNMT3a, Phosphorylation, Autophagy, PCa, Cell death, Crosstalk.

Running title: The Plk1/DNMT3a axis regulates autophagy in PCa.

*To whom correspondence should be addressed: Department of Toxicology and Cancer Biology, University of Kentucky, 1095 V.A. Drive, Lexington, KY 40536 Tel: 859-562-2006; Fax: 859-323-1059; Email: xiaoqi.liu@uky.edu.

Disclosure of Potential Conflicts of Interest: The authors declare no potential conflicts of interest.

# These authors contributed equally.

Supplementary Materials and Methods

High-throughput data preprocessing. Microarray Affymetrix Human Exon 1.0 ST Array data (.cel files) were processed and normalized by Robust Multi-array Average (RMA) algorithm [1]. TCGA-PRAD RNAseqV2 raw counts in gene level were normalized by total reads and then by gene length as RPKM (Reads Per Kilobase of transcript, per Million mapped reads) [2]. Then, the normalized transcript counts RPKM of an individual gene was corrected as a proportion of transcript read counts in the entire sample set by ratio (mean of group of housekeeping gene ACTB expression to current ACTB expression) to each sample. Log2-transformation normalized densities of targeted genes were used for further statistical analysis.

Construction of XDeath model

1) **Cell death pathway profiles and its network ontology construction:** We collected 29 cell death pathways with 2,698 genes from Gene Set Enrichment Analysis (GSEA) MsigDB C2 databse across 8 cell death modes, including apoptosis, autosis, autophagy, necroptosis, ferroptosis etc. These 29 cell death pathways have 3,356,224 interactions of genes. 1,832 overlapping genes in TCGA and GEO datasets are identified. 123 out of 1,832 genes are reported as hallmark genes by Nomenclature Committee on Cell Death (NCCD) in 2018 and 271 of 1,832 genes are recommended as drug-gable targets by Drug Bank annotation (https://www.drugbank.ca/releases/latest). These 29 cell death pathways have 3,356,224 interactions between genes which will be used for the study.

2) **XDeath for modeling of signaling crosstalk in disease progression networks**: XDeath is a dynamic data-driven network-based model by graphical regression integration with density-clustering to infer the crosstalk pathways of cell death during multiple space variations, such as gleason score (GS), to seek the optimal cancer targets.

The systems biology model aims to disclose the molecular mechanism contributing to disease progression via dynamic co-expression networks. Transcriptome data is used to figure out overlapping genes involved in the pathways of 8 cell death modes in PCa. Patients were categorized upon GS. Gene enrichment variations could be analyzed for changes across disease progression once normalized (GS5) via tumors (GSj, j=6, 7, 8, 9). Through the spearman correlation analysis, gene-gene interactions are restructured in each group with differential GS status to seek trend of enrichment genes or pairs of genes associated with disease progression.

The trends signify the genes dramatically varied across groups with different GS and these genes can be mapped back to the pathways in which they are involved and therefore, cell death modes as well. We then calculate the crosstalk enrichment score between every two pathways and rank the scores. Once high co-expression interactions are identified, the variations across GS statuses can be mapped. In this case, disease progression can be verified through two factors: gene/node enrichment analyses and interaction/edge enrichment analyses. For example, in patients with GS 5, all nodes and edges will be neutral because they are benign. However, in the patients with GS 6-9, node color and edge thickness may vary depending on the changes of gene expression. The enriched networks of specific GS statuses can then be traced back to their corresponding pathways. These pathways are then visually condensed. The application of enrichment scores in specific pathways identifies the cell death modes in consistent to signify likelihood of crosstalk. Significant crosstalk events are then identified by calculating the total crosstalk enrichment score between every two pathways across different cell death modes and will outline hallmark or target genes. In significant crosstalk events, these genes will have high levels of co-expression that can be traced back to the density cluster. Therefore, the targets genes are now considered druggable candidates for novel therapies.

3) **XDeath algorithm flowchart**: The XDeath could precisely identify the crosstalk variation of cell death signaling pathways by five steps. The first step is to preprocess high-throughput data and valid gene/pathway pairs. It includes the gene expression profiles' normalization. Second, we re-constructed a co-expression network by Spearman correlation in each state. Third, we scored weights for nodes and edges variations. The node variation is the differential gene expression calculations and the variation in edges is the difference of tumors verses its adjacent normal gene expression profile. Fourth, density clustering calculation of weight scoring nodes/edges is used to detect disease progression enrichment. Fifth, Kappa statistic test is used to assess graphical sets significance.

Cell-death pathway profiling and network ontology. Step 1. Differentially expressed genes (DEG) were calculated between tumor samples and normal samples with different GS status. **Step 2.** Gene set enrichment analysis (GSEA) [3] was performed to identify pathway activation in PCa by comparing normal versus tumor samples with different GS. GSEA software of 6.2 version of the Molecular Signatures Database (MSigDB) C2-Canonical pathways (CP) gene sets was used to identify the pathway enrichment of DEGs in PCa (normal versus tumor). All

pathway normalized enrichment scores (NES) in each GS were ranked. All ranks of NES were organized and used for the next step calculation. **Step 3**. Identification of hallmark genes and enriched pathways of disease progression. A Spearman correlation model $r = corr(x, y)$ was used to calculate the correlation coefficient r and its p-value to variation of genes or pathways associated with GS increase. Here, x is the gene differential expression degree between tumor and normal tissues, such as *fold change*, and y is the value of GS. For pathways, x is the value of NES and y is the value of GS. A positive correlation means the gene/pathway is activated with GS increasing and contributes to PCa progression. The disease progression hallmark genes and enrichment pathways were obtained by ranking these correlation coefficients. A graph Laplacian matrix M was constructed, where row and column are genes/pathways, and the value in the middle denotes edge correlation coefficient r. **Step 4.** Quantification of crosstalk revealed the synergistic relationship of genes. With our Fast and Furious Bayesian Network (FFBN) inference model [4], we constructed a whole genome regulation network to determine the causal direction of edges in late-stage PCa. Within the network, crosstalking genes are defined as those that are incident on interactions between Pair of Genes' first neighbor gene Sets (IPGSs), which include the overlapping genes and the interaction edges (**Fig. S1C**). The effect of crosstalk on the interaction density between two subnetworks was quantified. The decrease in interaction density after crosstalk nodes and edge IPGSs were removed from two merging subnetworks was termed *ΔD*. In this way, exclusivity on gene-gene pair interaction network was tested by Fisher's exact test in the analysis of the contingency table to the number of four status from two sub-networks within each GS status (**Supplementary Table 2**). **0** means no overlapping to edges, **1** means overlapping to edges.If the marginal totals N, K, M are known in the contingency table, only a single degree of freedom X is left. Fisher's exact test showed that the probability of obtaining any such set of values X was given by the hypergeometric distribution:

$$p(X \leq d) = F(X|N, K, M) = \sum_{X=0}^{d} \frac{\binom{K}{X}\binom{N-K}{M-X}}{\binom{N}{M}} = \sum_{X=0}^{d} \frac{(N-K)!K!(N-M)!M!}{a!b!c!X!}$$

where the total number of edges is N, X is the number of overlapping edges in two modules (subnetworks). $\binom{K}{X}$ is the binomial coefficient and the symbol ! indicates the factorial operator. Empirical p-value of ΔD is calculated by use of a null distribution of ΔD of crosstalk genes controlled by one million randomly selected gene pairs from the input contingency table, referring to algorithm Binox [5]. Pairs of gene score from IPGSs were ranked from top to bottom

in ascending statistical significance. The top-ranked pairs of genes were defined as potential co-targeted genes. Here, the crosstalk effect between Plk1 and DNMT3a was ranked as the top 3. We therefore believe that co-targeting Plk1 and DNMT3a offers a potential therapeutic strategy for patients with advanced PCa. **Step 5**. Crosstalk of molecular mechanisms between pairs of genes on network was revealed by the shortest path Dijkstra's algorithm on network Laplacian matrix *M*. The main idea is to seek the shortest connection from gene A to gene B such that the total sum of the edge (crosstalk of pairs of genes) weights is minimum (**Fig. S1D**). As such, we can identify a set of non-redundant shortest paths for the pairs of genes by one million random selection. The statistical significance of the average length of multiple short paths that connect gene A to gene B can be calculated by a chi-squared test (x^2 test). The significant short paths support the identified crosstalk of mechanisms of paired gene A and gene B on the graph.

Step 6. Analysis of upstream regulators of crosstalking genes. The goal of upstream regulator analysis is to identify molecules upstream of these crosstalking gene sets that potentially explain the observed expression changes [6]. Here, Ingenuity Pathway Analysis (IPA) software (http://www.ingenuity.com) was used to score regulatory networks of upstream genes based on a large-scale causal gene/protein network derived from its Ingenuity Knowledge Base. The *p*-values of these crosstalking genes with DEGs (normal versus tumor) were subjected to IPA analysis. The IPA of upstream regulators defines an overlap *p*-value that measures enrichment of network-regulated genes in the dataset, as well as an activation *Z*-score that can be used to find likely regulating molecules based on a statistically significant pattern matching up-regulation, and to predict the status (either activated or inhibited) of a putative regulator as well.

Protein-Protein Interaction (PPI) analysis. The human PPI data was downloaded from the search tool for the retrieval of interacting genes/proteins (STRING) database (https://string-db.org/). In total, 19,472 proteins were included and 11,873,250 PPIs were obtained.

Differential expression analysis. This was carried out for all genes in tumors of each GS status and adjacent normal samples. All data analysis was performed with R software with R 'limma' package being used for DEG analysis of microarray GSE21034 data. To TCGA-PRAD RNAseqV2 data, EdgeR tool was used to carry out differential analysis where raw counts were the input. Genes with reads count smaller than 10 in total samples were filtered out, and genes with absolute value to fold change >2 and adjusted p value<0.05 were considered as significantly differential expression.

Pathway enrichment analysis. GSEA software [3] was used for pathway gene set enrichment analysis. Gene expression profiles and associated with phenotypes input into GSEA, parameters setting is to select MSigDB v6.2 database C2 Canonical pathways (CP) set as reference pathways background for the pathway enrichment analysis.

Gene regulation network by Bayesian causal inference. FFBN inference model [4] was used to construct a whole genome regulation network with significantly fewer model configuration rules to determine the causal direction of edges, especially to tumors in a specific GS status.

Network analysis. The layout of disease network was grouped by the classification of GS, and node degree is the number of interactions for each node in the global protein-protein network. Upstream regulator analysis in network was conducted by IPA software. These significant genes are entered into IPA to seek their key upstream regulatory genes based on a large-scale causal gene network derived from IPA Knowledge Base. The software Cytoscape [7] was used to conduct network visualization.

Network interaction of cell death and searching engineer. We constructed a visualizing system platform CrosstalkDeathDB to satisfy user-specific needs, with free access by the link https://pcm2019.shinyapps.io/LijunLab-CD-pathway/. CrosstalkDeathDB platform was designed to tailor gene-gene and subnetwork-subnetwork relationships within all the existing cell-death modes.

Correlation variation of mRNA-methylation. Differentially methylated CpG sites (DMCs), differentially methylated regions (DMRs), and DEGs were identified by two-sample t-test. The relationship between the two omics was further analyzed by Pearson correlation analysis. In addition, significant DMRs and CpG site co-methylation, co-gene expression and co-methylation and gene expression network (WCCN) was constructed, followed by Spearman correlation analysis to mRNA, methylation and an integrated analysis of DNA methylation and gene expression. The Cheng lab has developed a pattern recognition and match computational method, called 3MCdiscovery, for matching these three networks cluster patterns of DMRs, DMCs and their correlation mRNA-methylation and seek methylation-mRNA variation patterns and drive DNA methylation variation with PCa disease progression.

Cell culture and transfection. PC3 cells were cultured in ATCC-formulated F-12K medium supplemented with 10% (v/v) fetal bovine serum (FBS) at 37°C in 5% CO_2. DU145 cells were cultured in Dulbecco's Modified Eagle Medium with 10% FBS. Cells were transiently

transfected with plasmid DNA with Lipofectamine 2000 transfection reagent from Invitrogen. Cells stably expressing different forms of DNMT3A (WT, S390A, S393A) were obtained upon selection with 0.8 mg/ml G418 (Sigma) for 2 weeks.

Reagents and plasmid DNA. MG132, cycloheximide and nocodazole were purchased from Sigma, BI2536, rapamycin and 5-Aza were obtained from Selleckchem, the human DNMT3a and mGFP-RFP-LC3 plasmids were ordered from Addgene, and the site-directed mutagenesis kit, used to generate the specific mutation, was purchased from Agilent Technologies. Antibodies against DNMT3A, β-actin, GAPDH, p-H3, Cyclin D1/D2, myc, HA, LC A/B, p62, ULK1, p-S6, S6, p-ULK-S555, p-ULK-S757, p-AMPK-T172, p-AMPK-S485, ULK1, AMPK, Cleaved PARP, and PCNA were purchased from Cell Signaling Technology (CST). Antibodies against Flag, ubiquitin, Plk1 and p-DNMT3a were purchased from sigma, Santa Cruz, Millipore, and Abcam, respectively.

Immunofluorescence (IF) staining. Cells were incubated with antibodies against Ki67 (catalog no 9449; CST) and cleaved-caspase 3 (catalog no 9661; CST) for 1 hour at room temperature, followed by incubation with secondary antibody and 4' 6-diamidino-2-phenylindole (DAPI; Sigma) for 1 hour.

Immunochemistry (IHC) staining. After tumors were fixed with 10% Formalin overnight, slides were incubated with first antibodies for overnight at 4 °C, followed by incubation with secondary antibodies for I hour at room temperature and development of the colored product of the enzyme with the appropriate chromogen (DAB substrate kit, SK-4100, Vector).

Scoring of immunochemistry staining in human tissue microarrays

Human prostate cancers were harvested and immunochemistry stained. Tumors were graded on a total score of score for staining proportion (from 0 to 5) plus score for staining intensity (from 0 to 3). 1) Score for proportion: 0= no staining; 1= ≥0% and ≤1% nuclei staining; 2= ≥1% and ≤10% nuclei staining; 3= ≥10% and ≤33% nuclei staining, and 4= ≥33% and ≤66% nuclei staining; 5= ≥66% and ≤100% nuclei staining, and 2) Score for intensity: 0= no staining; 1= weak staining; 2= moderate staining; and 3=strong staining. Maximum score =8. Score≤2 means negative. Score >2 means positive.

Immunoblotting (IB) and immunoprecipitation (IP). Cell lysates were prepared in TBSN buffer (20 mM Tris, pH 8.0, 150 mM NaCl, 0.5% Nonidet P-40, 5 mM EGTA, 1.5 mM EDTA, 20 mM p-nitrophenyl phosphate). For IP, lysates were incubated with various antibodies in

TBSN buffer at 4°C overnight, followed by 3 washes with TBST buffer plus 500 mM NaCl and 3 additional washes with TBST buffer and 150 mM NaCl. For IB, filters were incubated with specific antibodies at 4°C overnight, followed by an additional incubation with the secondary antibody for 1 hour.

MTT and colony formation. For the MTT assay, cells (2×10^3) were seeded into 96-well plates, incubated for 0-6 days, and stained with MTT dye (Sigma, MO), followed by measurement of absorbance at 490 nm. For the colony formation assay, cells (1×10^3) were seeded into 6-well plates, cultured for 7 to 12 days, fixed with paraformaldehyde, and stained with Coomassie brilliant blue.

Supplementary Figures and Figure Legends

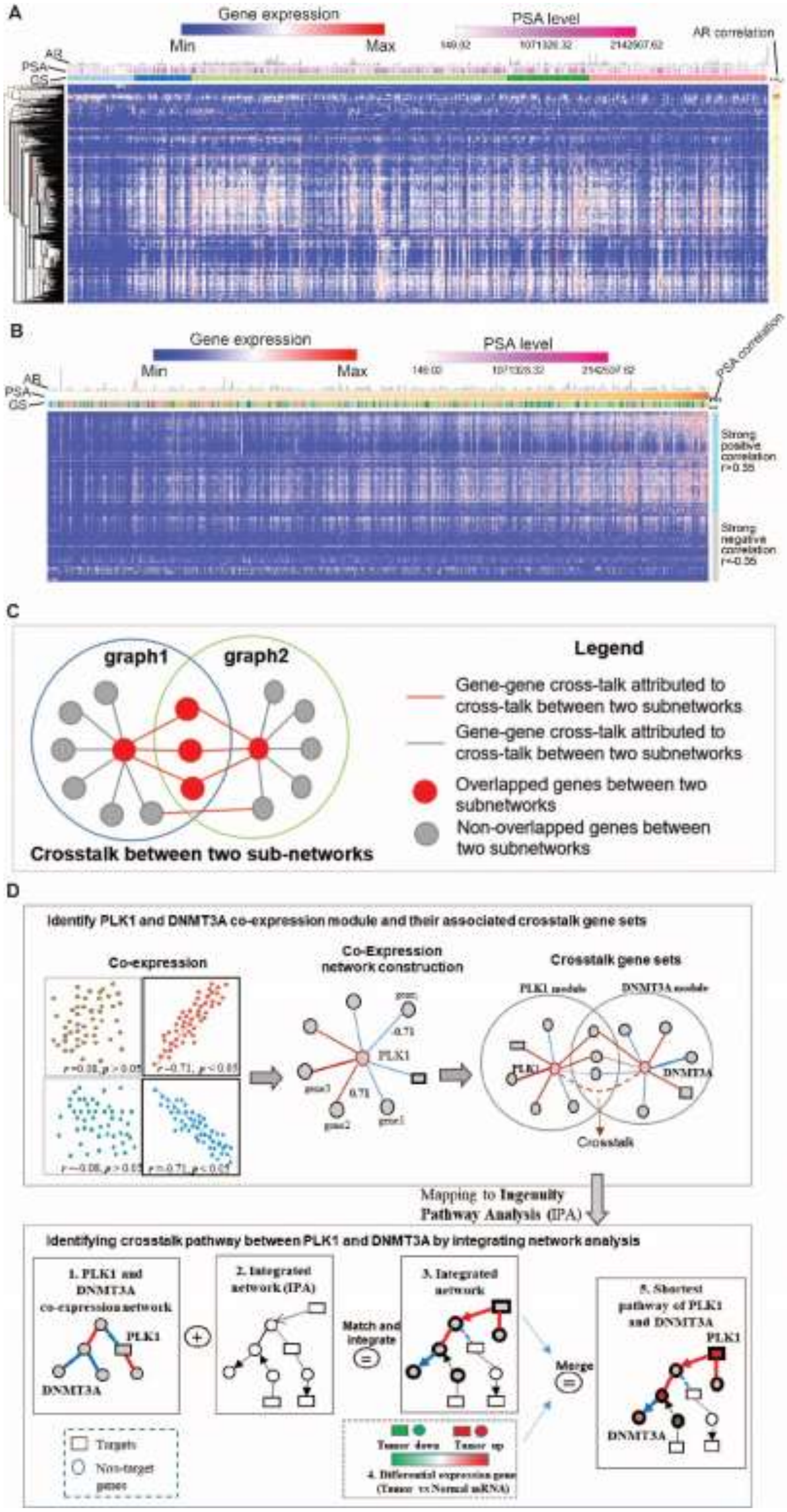

Supplementary Figure 1. **Integrative hierarchical analysis of PCa progression. A**, Heatmap

of gene expression in patients with various levels of PSA. **B**, Heatmap of gene expression in the context of correlation between GS and PSA levels. **C,** Crosstalk of subnetworks. The overlapping region between the two subnetworks reveals the common genes and interacting edges. **D**, Molecular mechanism of pathway interactions. The upper panel identifies Plk1 and DNMT3a co-expressing modules and their associated crosstalk gene sets, the lower panel identifies the crosstalk pathways between Plk1 and DNMT3a by integrating network analysis.

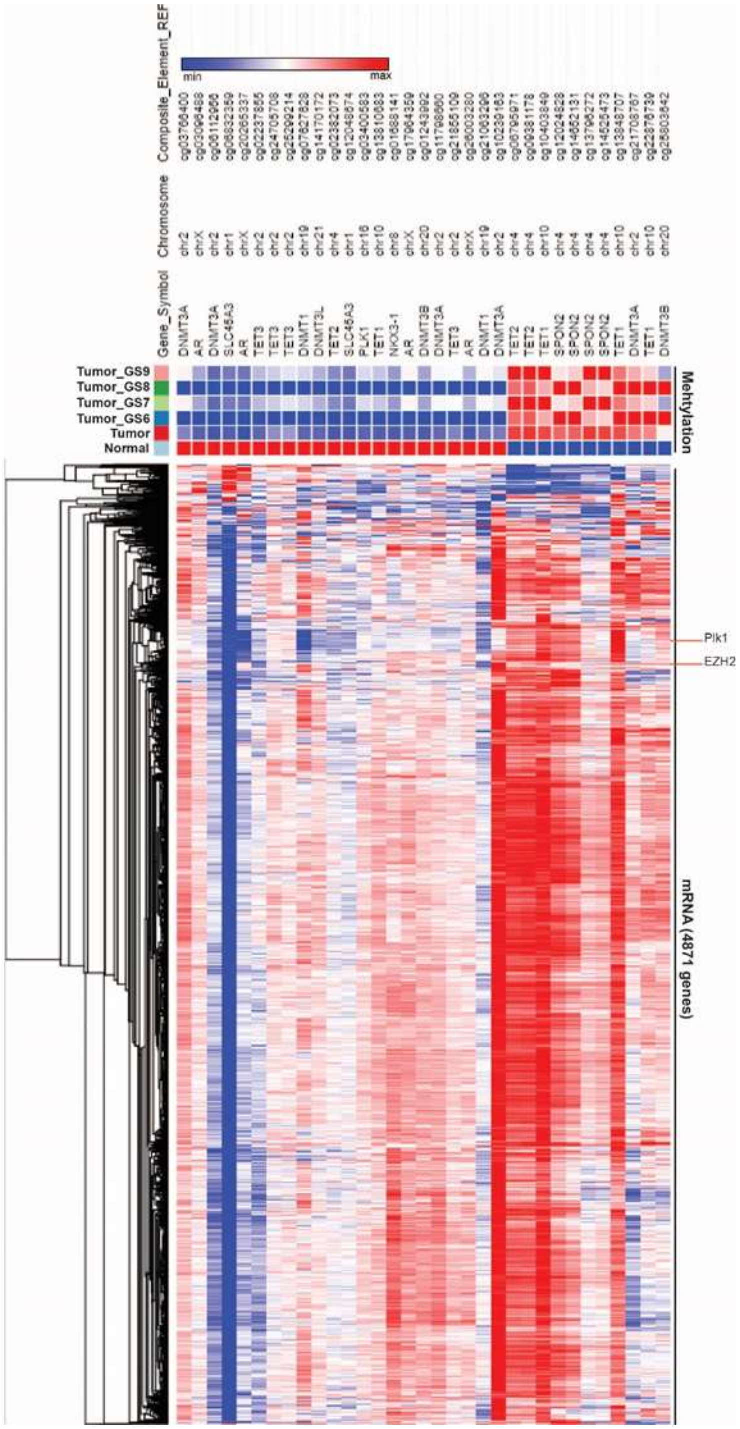

Supplementary Figure 2. Average correlation analysis for methylation of targeted genes and mRNA of 4871 genes in 533 PCa patients from TCGA dataset.

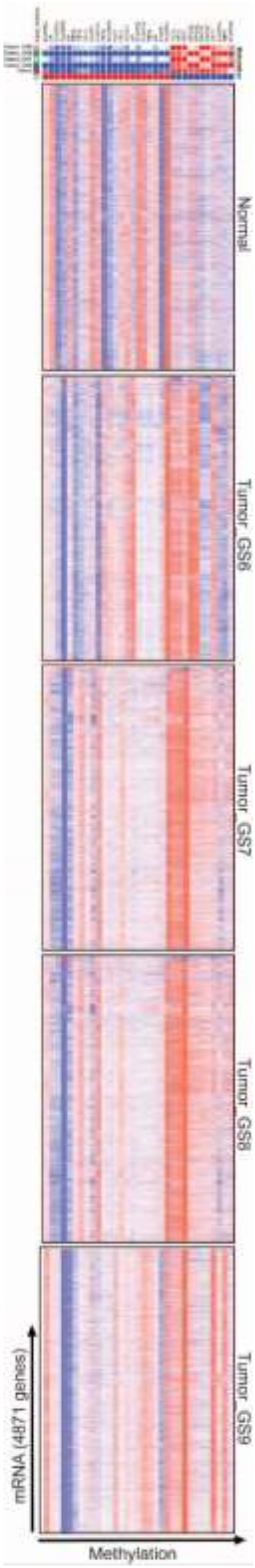

Supplementary Figure 3. Methylation and gene expression profiles and associations in PCa patients with various GS status.

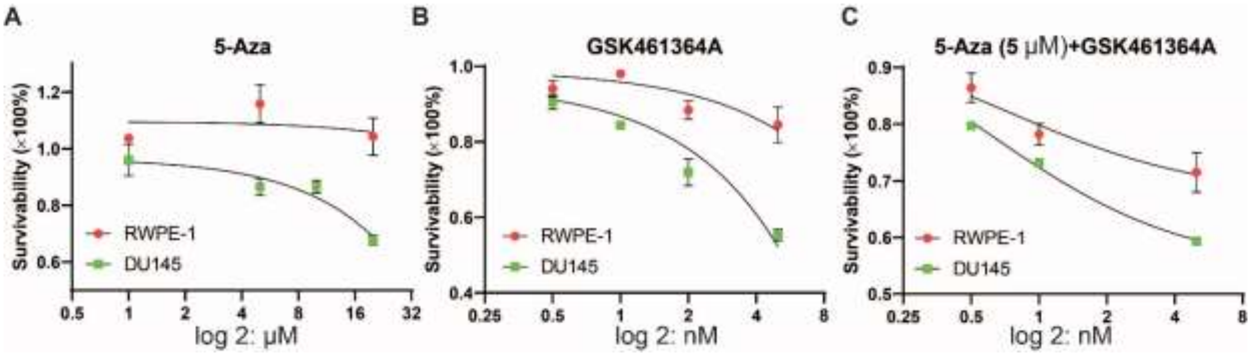

Supplementary Figure 4. Cell viability assays in RWPE-1 and DU145 cells treated with 5-Aza and/or GSK461364A. A, RWPE-1 and DU145 cells were treated with different dose of 5-Aza for 24 hours, followed by MTT assay. **B**, RWPE-1 and DU145 cells were treated with different dose of GSK461364A for 24 hours, followed by MTT assay. C, RWPE-1 and DU145 cells were treated with 5 μM 5-Aza plus different dose of GSK461364A for 24 hours, followed by MTT assay.

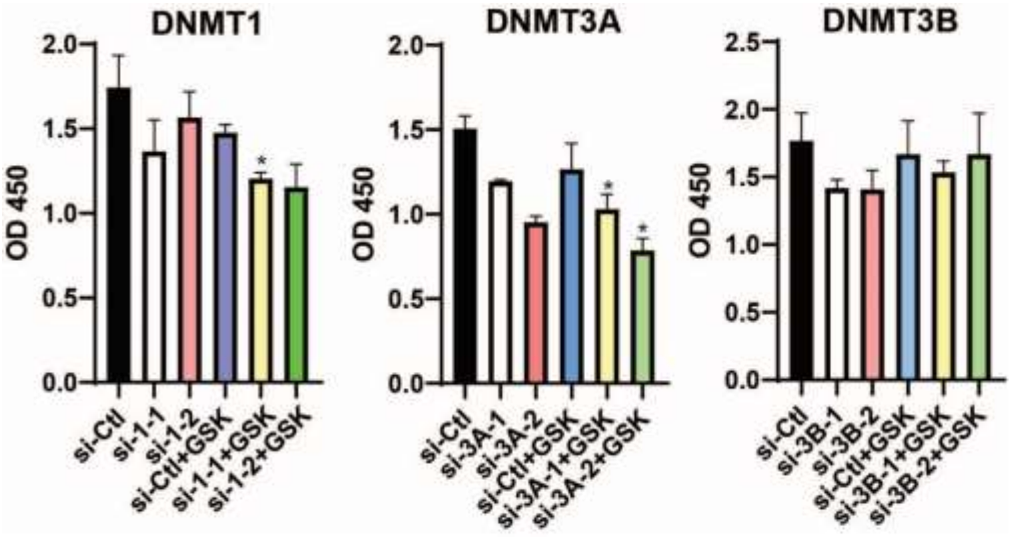

Supplementary Figure 5. Cell viability assays in cells depleted of DNMT1, DNMT3a and DNMT3b plus GSK461364A treatment.

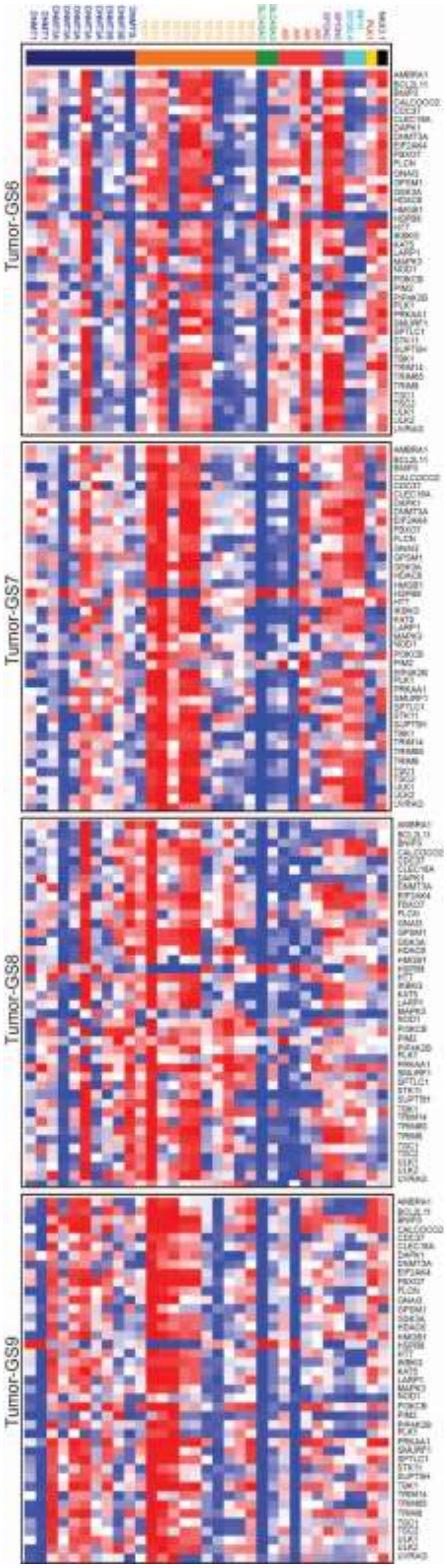

Supplementary Figure 6. Heatmap for correlation between methylation and transcription of autophagy-related genes in PCa samples with different GS.

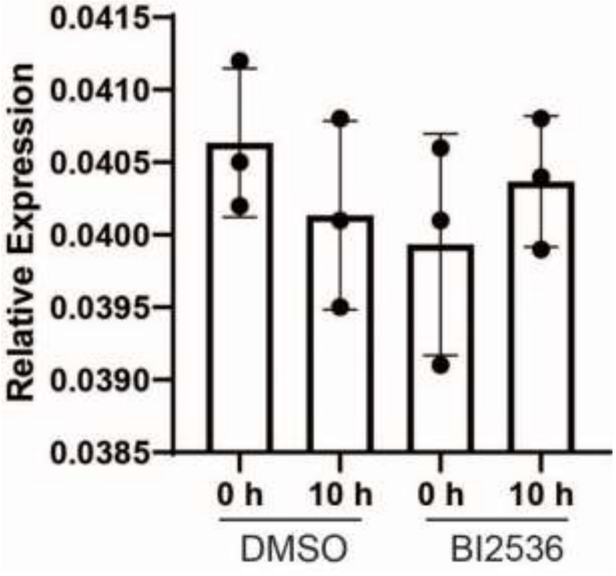

Supplementary Figure 7. mRNA levels of DNMT3a in DU145 cells treated with or without BI2536 for 0 and 10 hours after the double thymidine blocking.

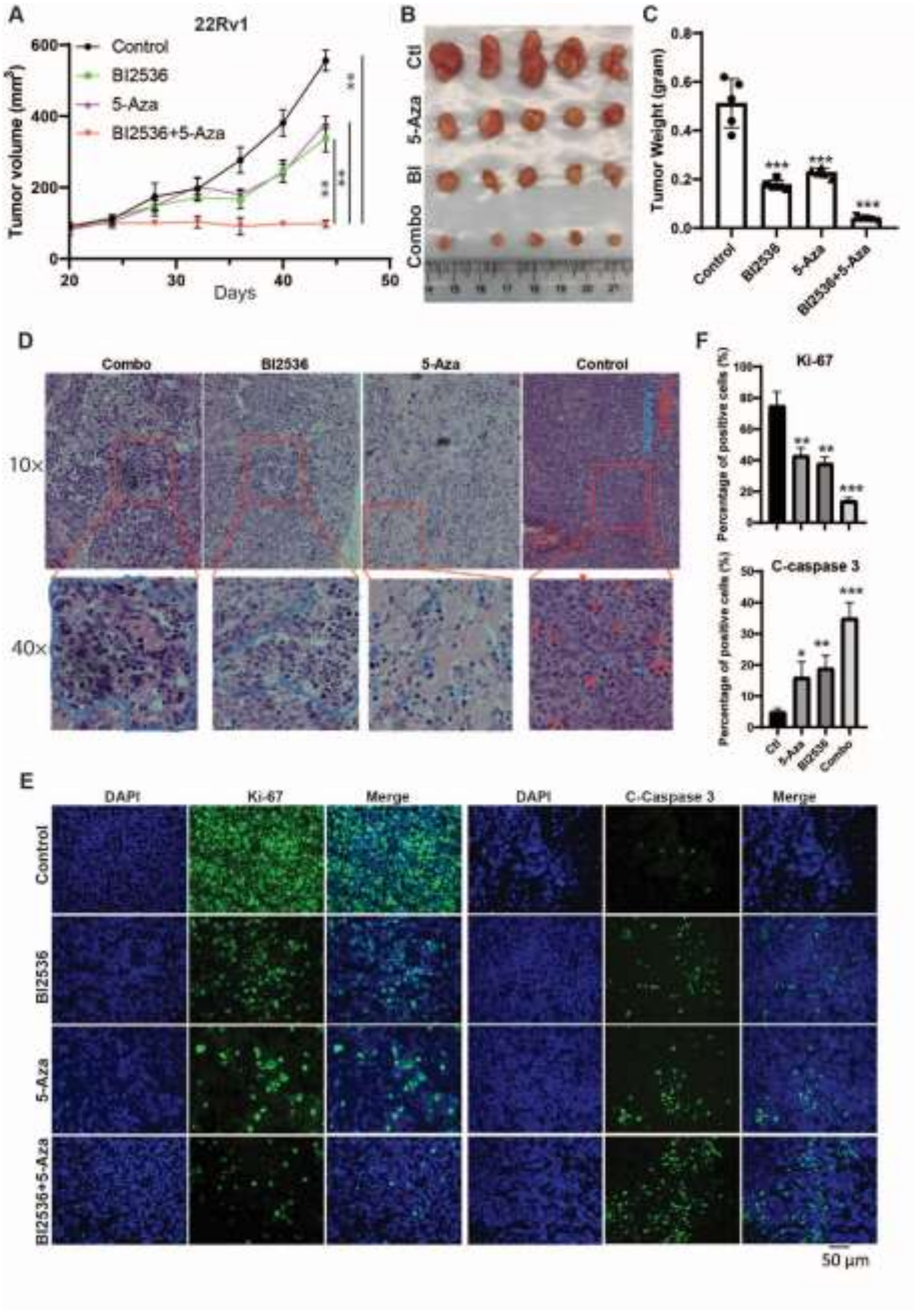

Supplementary Figure 8. Inhibition of Plk1 and DNMT3a suppress DU145-derived xenograft tumor growth synergistically. **A**, Nude mice were inoculated with 22Rv1 cells (2.5 x 10^5), treated with BI2536, 5-Aza or both, followed by measurement of tumor size every 4 days (means ± SE; n=5). **B**, Tumors at the end of the study. **C**, Wet weights of the tumors. **D**, H&E staining of the tumors. (Red arrows: mitotic cells, blue arrows: apoptotic cells). **E**, IF staining against Ki-67 and Cleaved caspase 3. **F**, Quantification of E. *, 0.01<p<0.05, **, 0.001<p<0.01, ***, p<0.001.

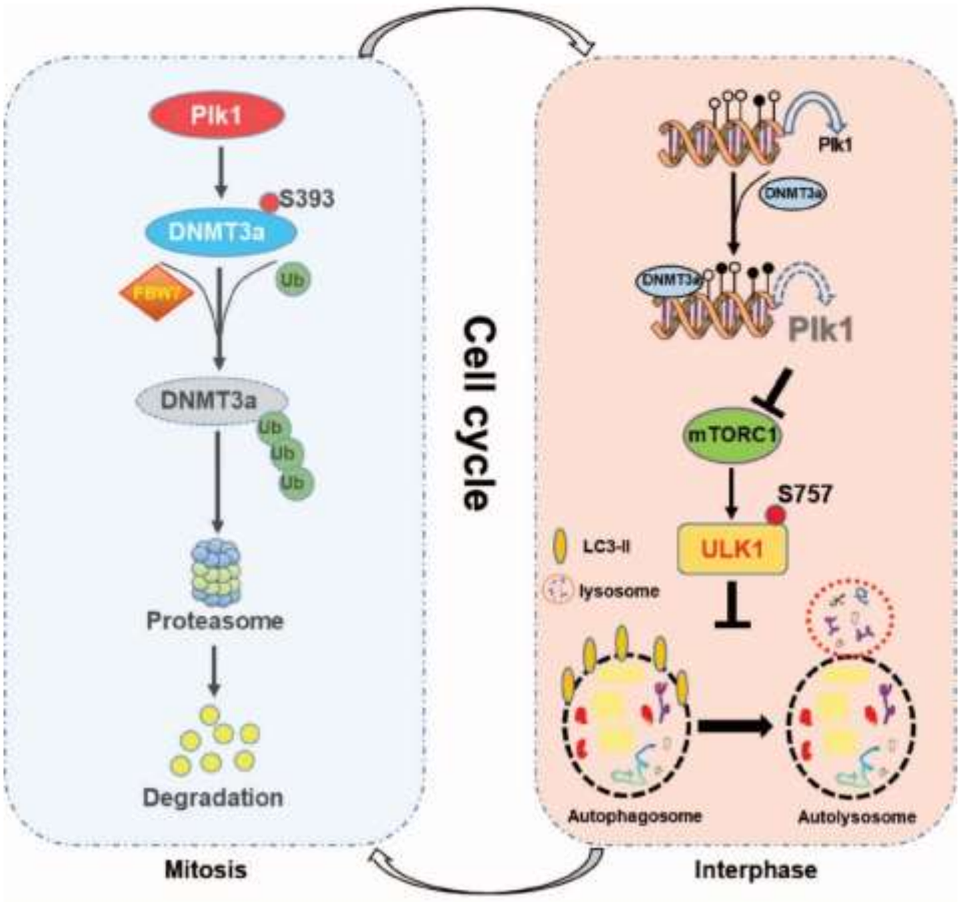

Supplementary Figure 9. A working model depicting mutual regulations between Plk1 and DNMT3a during cell cycle in PCa cells.

In interphase, elevation of DNMT3a results in hypermethylation of Plk1 promotor region and suppression of transcription of Plk1. When DNMT3a is inhibited by 5-Aza, suppression of Plk1 expression will be released due to hypomethylation of its promoter. In addition to the mitotic role of Plk1 as a cell cycle related kinase, the regulation of its non-mitotic signaling plays important roles in cancer cells as well. As illustrated, Plk1 promotes autophagy independent of its mitotic effects whereas DNMT3a inhibits that autophagy by suppressing expression of Plk1. In that case, inhibition of DNMT3a by 5-Aza promotes autophagy by elevating Plk1. Elevation of Plk1 provides targets for Plk1 inhibitor treatment. In mitosis, Plk1 inhibits DNMT3a via phosphorylation at S393. Plk1 directly phosphorylates DNMT3a at S393 leading to the degradation of DNMT3a. In this case, inhibition of Plk1 by BI2536 stabilizes DNMT3a by dephosphorylation at S393 resulting in further inhibition of autophagy. In sum, such novel mutual negative regulations between Plk1 and DNMT3a provide a rationale for combination treatment with inhibition of Plk1 and DNMT3a in advanced PCa.

Supplementary Table 1. Information of PCa specimens in two datasets

Dataset ID	***Benign prostate tissues***	***Hormone-sensitive***		***CRPC***		***Sample size***
		**GS <=6**	**GS 7**	**GS 8**	**GS>=9**	**Total**
TCGA	52	45	248	64	141	550
GSE21034	29	80	50	10	10	179

Note Abbreviations: TCGA: The Cancer Genome Atlas; GDCDP: Genomic Data Commons Data Portal; GEO: Gene Expression Omnibus; GS: Gleason Score.

Supplementary Table 2. Crosstalk density calculation to overlapped genes and edges

Contingency table		Subnetwork 1		Row total
		**0**	**1**	
Subnetwork 2	**0**	a	b	$a+b(=N-K)$
	**1**	c	$d(=X)$	$c+d\ (=K)$
Column total		$a+c(=N-M)$	$b+d(=M)$	$a+b+c+d(=N)$

Supplementary Table 9. 7 pairs of genes and 15 single genes are recommended as druggable targets to treat advanced prostate cancer

ID	Gene_A	Gene_B	Trend score	Drugs
**1**	PSMB2	PSMB5	0.357486018	
**2**	MAPK8	PARP1	0.501335984	
**3**	PARP1	SLC25A5	0.553348706	
**4**	POLD1	PHB	0.539458079	
**5**	AURKA	MELK	0.585705236	
**6**	PARP1	HDAC1	0.61631213	
**7**	**PLK1**	**DNMT3A**	0.512705236	
**8**	HPRT1		0.9654064	6-mercaptopurine, thioguanine, azathioprine, cyclophosphamide/cytarabine/6-mercaptopurine, 6-mercaptopurine/prednisone, 6-mercaptopurine/prednisone/thioguanine, 6-mercaptopurine/methotrexate/tretinoin, daunorubicin/etoposide/6-mercaptopurine/mitoxantrone/
**9**	AURKB		0.8356063	barasertib, tozasertib, AT-9283, SNS 314, danusertib, TAK-901, GSK1070916, CYC 116, TTP607, AMG 900, ilorasertib, chiauranib
**10**	PLK1		0.73934	BI 2536, volasertib, GSK461364, rigosertib, MK1496, onvansertib, TAK-960, lipid encapsulated anti-PLK1 siRNA TKM-080301
**11**	PTK2		0.9971746	BI 853520, lorlatinib, CT-707, TPX-0005
**12**	F2R		0.960608	chrysalin, vorapaxar, PAR1 inhibitor, argatroban, bivalirudin
**13**	LIMK1		0.9176321	dabrafenib, encorafenib, dabrafenib/trametinib, cetuximab/encorafenib, dabrafenib/panitumumab, dabrafenib/trametinib/vemurafenib, dabrafenib/panitumumab/trametinib, binimetinib/encorafenib, dabrafenib/ipilimumab/trametinib
**14**	CENPE		0.9981194	GSK923295
**15**	KIF11		0.8727248	ispinesib, AZD4877, MK-0731, ARRY-520, SB-743921, litronesib, 4SC-205, ALN-VSP02
**16**	TOP2A		0.9217149	novobiocin, etoposide, teniposide, CPI-0004Na, becatecarin, elsamitrucin, AQ4N, elomotecan, tafluposide, fleroxacin, cyclophosphamide/epirubicin/5-fluorouracil, cytarabine/daunorubicin, finafloxacin, hydrocortisone/mitoxantrone, mitoxantrone/prednisone, c
**17**	MELK		0.9367354	OTS167
**18**	PARP1		0.9323572	poly ADP ribose polymerase 1 inhibitor, veliparib, rucaparib, olaparib, niraparib, talazoparib, E7449, ABT-767, CEP-9722, fluzoparib, SC10914, simmiparib, INO-1001, [18F]fluorthanatrace
**19**	DHFR		0.9358572	pyrimethamine, trimethoprim, iclaprim, proguanil, methotrexate/ofatumumab, methotrexate/sirolimus/tacrolimus, pralatrexate, abatacept/methotrexate, infliximab/methotrexate, piritrexim, methotrexate/rituximab, golimumab/methotrexate, cisplatin/doxorubicin/
**20**	AURKA		0.9146267	tozasertib, MLN8054, alisertib, AT-9283, SNS 314, danusertib, MK 5108, CYC 116, TTP607, AMG 900, ilorasertib, TAS-119
**21**	MAPK3		0.9751645	ulixertinib, LY3214996, KO-947, ASN007, ASTX029
**22**	BRAF		0.9978444	vemurafenib, dabrafenib, regorafenib, bortezomib/sorafenib, dexamethasone/lenalidomide/sorafenib, bevacizumab/sorafenib, encorafenib, dabrafenib/trametinib, CEP-32496, PLX8394, cytarabine/idarubicin/sorafenib, 5-azacytidine/sorafenib, decitabine/sorafenib

Supplementary Table Legends

Supplementary Table 3.1 Annotation of all samples of PCa patients in **TCGA**, including demographic information, GS, and survival time etc.

Supplementary Table 3.2 Annotation of all samples of PCa patients in **GSE21034**, including demographic information and GS.

Supplementary Table 4. The top 400 disease genes related to GS in **TCGA**. Expressions of all these genes have a significant difference between tumor and adjacent normal with a strong correlation with GS.

Supplementary Table 5. The gene expression data of top 400 genes related to GS in **TCGA**.

Supplementary Table 6.1. The differential expression rank of genes with different GS in comparison of tumor to adjacent normal in **TCGA**.

Supplementary Table 6.2. The differential expression rank of genes with different GS in comparison of tumor to adjacent normal in **GSE21034**.

Supplementary Table 7. The gene expression data of top 1,000 genes with the largest standard variation in **TCGA**. The average Pearson correlations of these 1,000 gene expression to PSA, AR and GS are 0.35, 0.25 and 0.07, respectively.

Supplementary Table 8.1 The pathway enrichment score (NES, Normal Enrichment Score) of tumor versus adjacent normal in **TCGA**.

Supplementary Table 8.2 The pathway enrichment score (NES, Normal Enrichment Score) of cell death pathway of tumor versus adjacent normal in **TCGA** with different GS.

Supplementary Table 8.3 The pathway enrichment score (NES, Normal Enrichment Score) of tumor versus adjacent normal in **GSE21034**, and cell death mode annotation with different GS.

Supplementary Table 10. The potential co-targets of pairs of pathways during PCa progression by Binox analysis.

Supplementary Table 11. The cross talk molecular mechanism of Plk1 and DNMT3a on a differential network. The comparison is between adjacent normal and tumors in TCGA by Pearson Correlation to detect the interaction changes of co-expression gene regulation.

Supplementary Table 12. The cross talk molecular mechanism of Plk1 and DNMT3a on a protein-protein interaction network by IPA upstream casual analysis.

References

[1] R. A. Irizarry, B. Hobbs, F. Collin, Y. D. Beazer-Barclay, K. J. Antonellis, U. Scherf, T. P. Speed, *Biostatistics*.**2003**, *4*, 249.

[2] C. Evans, J. Hardin, D. M. Stoebel, *Brief Bioinform*.**2018**, *19*, 776.

[3] A. Subramanian, P. Tamayo, V. K. Mootha, S. Mukherjee, B. L. Ebert, M. A. Gillette, A. Paulovich, S. L. Pomeroy, T. R. Golub, E. S. Lander, J. P. Mesirov, *Proc Natl Acad Sci U S A*.**2005**, *102*, 15545.

[4] E. Liu, G. Kinnebrew, J. Li, P. Zhang, Y. Zhang, L. Cheng, L. Li, *IEEE/ACM Trans Comput Biol Bioinform*.**2019**.

[5] C. Ogris, D. Guala, T. Helleday, E. L. Sonnhammer, *Nucleic Acids Res*.**2017**, *45*, e8.

[6] A. Kramer, J. Green, J. Pollard, Jr., S. Tugendreich, *Bioinformatics*.**2014**, *30*, 523.

[7] M. Kohl, S. Wiese, B. Warscheid, *Methods Mol Biol*.**2011**, *696*, 291.
